# Predatory protists impact plant performance by promoting plant growth-promoting rhizobacterial consortia

**DOI:** 10.1093/ismejo/wrae180

**Published:** 2024-09-23

**Authors:** Sai Guo, Stefan Geisen, Yani Mo, Xinyue Yan, Ruoling Huang, Hongjun Liu, Zhilei Gao, Chengyuan Tao, Xuhui Deng, Wu Xiong, Qirong Shen, George A Kowalchuk, Rong Li

**Affiliations:** The Sanya Institute of the Nanjing Agricultural University, Jiangsu Provincial Key Lab of Solid Organic Waste Utilization, Jiangsu Collaborative Innovation Center of Solid Organic Wastes, Educational Ministry Engineering Center of Resource-saving fertilizers, College of Resources and Environmental Sciences, Nanjing Agricultural University, Nanjing, Jiangsu Provice 210095, P. R. China; Laboratory of Nematology, Wageningen University, Wageningen 6700 AA, the Netherlands; The Sanya Institute of the Nanjing Agricultural University, Jiangsu Provincial Key Lab of Solid Organic Waste Utilization, Jiangsu Collaborative Innovation Center of Solid Organic Wastes, Educational Ministry Engineering Center of Resource-saving fertilizers, College of Resources and Environmental Sciences, Nanjing Agricultural University, Nanjing, Jiangsu Provice 210095, P. R. China; The Sanya Institute of the Nanjing Agricultural University, Jiangsu Provincial Key Lab of Solid Organic Waste Utilization, Jiangsu Collaborative Innovation Center of Solid Organic Wastes, Educational Ministry Engineering Center of Resource-saving fertilizers, College of Resources and Environmental Sciences, Nanjing Agricultural University, Nanjing, Jiangsu Provice 210095, P. R. China; The Sanya Institute of the Nanjing Agricultural University, Jiangsu Provincial Key Lab of Solid Organic Waste Utilization, Jiangsu Collaborative Innovation Center of Solid Organic Wastes, Educational Ministry Engineering Center of Resource-saving fertilizers, College of Resources and Environmental Sciences, Nanjing Agricultural University, Nanjing, Jiangsu Provice 210095, P. R. China; The Sanya Institute of the Nanjing Agricultural University, Jiangsu Provincial Key Lab of Solid Organic Waste Utilization, Jiangsu Collaborative Innovation Center of Solid Organic Wastes, Educational Ministry Engineering Center of Resource-saving fertilizers, College of Resources and Environmental Sciences, Nanjing Agricultural University, Nanjing, Jiangsu Provice 210095, P. R. China; Department of Research and Innovation, EUROstyle BV, Ecomunitypark 1, Oosterwolde 8431 SM, the Netherlands; Ecology and Biodiversity Group, Department of Biology, Institute of Environmental Biology, Utrecht University, Padualaan 8, Utrecht 3584 CH, the Netherlands; The Sanya Institute of the Nanjing Agricultural University, Jiangsu Provincial Key Lab of Solid Organic Waste Utilization, Jiangsu Collaborative Innovation Center of Solid Organic Wastes, Educational Ministry Engineering Center of Resource-saving fertilizers, College of Resources and Environmental Sciences, Nanjing Agricultural University, Nanjing, Jiangsu Provice 210095, P. R. China; The Sanya Institute of the Nanjing Agricultural University, Jiangsu Provincial Key Lab of Solid Organic Waste Utilization, Jiangsu Collaborative Innovation Center of Solid Organic Wastes, Educational Ministry Engineering Center of Resource-saving fertilizers, College of Resources and Environmental Sciences, Nanjing Agricultural University, Nanjing, Jiangsu Provice 210095, P. R. China; The Sanya Institute of the Nanjing Agricultural University, Jiangsu Provincial Key Lab of Solid Organic Waste Utilization, Jiangsu Collaborative Innovation Center of Solid Organic Wastes, Educational Ministry Engineering Center of Resource-saving fertilizers, College of Resources and Environmental Sciences, Nanjing Agricultural University, Nanjing, Jiangsu Provice 210095, P. R. China; The Sanya Institute of the Nanjing Agricultural University, Jiangsu Provincial Key Lab of Solid Organic Waste Utilization, Jiangsu Collaborative Innovation Center of Solid Organic Wastes, Educational Ministry Engineering Center of Resource-saving fertilizers, College of Resources and Environmental Sciences, Nanjing Agricultural University, Nanjing, Jiangsu Provice 210095, P. R. China; Ecology and Biodiversity Group, Department of Biology, Institute of Environmental Biology, Utrecht University, Padualaan 8, Utrecht 3584 CH, the Netherlands; The Sanya Institute of the Nanjing Agricultural University, Jiangsu Provincial Key Lab of Solid Organic Waste Utilization, Jiangsu Collaborative Innovation Center of Solid Organic Wastes, Educational Ministry Engineering Center of Resource-saving fertilizers, College of Resources and Environmental Sciences, Nanjing Agricultural University, Nanjing, Jiangsu Provice 210095, P. R. China

**Keywords:** predatory protists, plant growth-promoting rhizobacteria, rhizosphere microbial interactions, plant performance enhancement

## Abstract

Plant performance is impacted by rhizosphere bacteria. These bacteria are subjected to both bottom-up control by root exudates as well as top-down control by predators, particularly protists. Protists stimulate plant growth-promoting microbes resulting in improved plant performance. However, knowledge of the mechanisms that determine the interconnections within such tripartite protist–bacteria–plant interactions remains limited. We conducted experiments examining the effects of different densities of the predatory protist *Cercomonas lenta* on rhizosphere bacterial communities, specifically zooming on interactions between *Cercomonas lenta* and key bacterial taxa, as well as interactions among key bacterial taxa. We tracked rhizosphere bacterial community composition, potential microbial interactions, and plant performance. We found that *Cercomonas lenta* inoculation led to an average increase in plant biomass of 92.0%. This effect was linked to an increase in plant growth-promoting rhizobacteria (*Pseudomonas* and *Sphingomonas*) and a decrease in bacteria (*Chitinophaga*) that negatively impact on plant growth-promoting rhizobacteria. We also found evidence for cooperative enhancements in biofilm formation within the plant growth-promoting rhizobacterial consortium. *Cercomonas lenta* enhanced a plant growth-promoting rhizobacterial consortium colonization by promoting its cooperative biofilm formation in the rhizosphere, leading to a 14.5% increase in phosphate solubilization that benefits plant growth. Taken together, we provide mechanistic insights into how the predatory protist *Cercomonas lenta* impacts plant growth, namely by stimulating plant beneficial microbes and enhancing their interactive activities such as biofilm formation. Predatory protists may therefore represent promising biological agents that can contribute to sustainable agricultural practices by promoting interactions between the plant and its microbiome.

## Introduction

The rhizosphere is a narrow, but dynamic zone, in soils that is directly affected by plant roots [1]. Plant-released organic carbon compounds (e.g. root exudates) [2] serve as nutrients and energy sources (e.g. amino acids and simple sugars) for microorganisms (e.g. bacteria and fungi) [3, 4]. Thus, the rhizosphere is a hotspot in terms of the densities and activities of microbial populations as compared to the bulk soil [5]. In addition to plant-encoded strategies to overcome various biological and abiotic stresses from their surroundings [6], plants also rely on plant-associated microorganisms for soil nutrition utilization, disease suppression, and drought stress mitigation, thereby improving plant performance [2, 7, 8].

Bacteria are the most abundant microorganisms in the rhizosphere, forming an integral part of complex microbial consortia [9, 10]. Some of these rhizobacteria have been termed plant growth-promoting rhizobacteria (PGPR), as they can enhance plant performance by, for instance, facilitating nitrogen fixation, mineralizing phosphate, producing plant-beneficial hormones, and suppressing pathogens [11]. Biofilm formation represents an important trait involved in root colonization and the ability of PGPR to enhance plant growth [12, 13]. At the same time, plant pathogenic bacteria can enter and colonize the rhizosphere, thereby negatively impacting plant performance [14], and it has been shown that the composition of the bio-control bacterial community can impact the population densities of pathogens [15].

The assembly of bacteria in the rhizosphere is known to be influenced by root exudates, following a traditional bottom-up perspective [16–18]. In addition to the impact of plants, bacteria are also subjected to top-down control by soil predators, particularly predatory protists [19]. Predatory protists are the main component of soil protists, and play a keystone role in soil food webs [19]. Predatory protists enhance plant performance by increasing the abundances of plant-beneficial IAA-producing and pathogen-suppressive bacteria, and releasing nitrogen from bacteria that is then available for the plant [20, 21]. However, not all predatory protists seem to perform the same functions. One of the most abundant genera in soil that has repeatedly been shown to be linked to plant growth and health is *Cercomonas* [22–24]. Yet, the mechanisms underlying the interconnections in the protist–bacteria–plant triangle remain largely unknown.

To investigate the mechanisms underlying the interconnections in the protist–bacteria–plant triangle, we first examined the impact of inoculation with different densities of the predatory protist *Cercomonas lenta* (*C. lenta*) on the bacterial community, including PGPR, and these data were linked with measures of plant biomass, using cucumber as a model plant. We then investigated potential cooperative interactions (e.g. biofilm formation) leading to a plant growth-promoting rhizobacterial consortium and plant growth-promoting functional capability of the consortium as well as its links with changes in plant performance. We further performed subsequent mesocosm experiments to validate the functional importance of the plant growth-promoting rhizobacterial consortium induced by the predatory protist *C. lenta*. We hypothesized that (i) *C. lenta* would impact the rhizosphere bacterial community leading to the enrichment of some plant-beneficial bacteria (e.g. PGPR) at the expense of other bacterial taxa (e.g. potential PGPR antagonists), thereby enhancing plant performance, and that (ii) the plant growth-promoting effects of the protist-induced rhizobacterial consortium are due to enhanced biofilm formation and plant growth-promoting abilities, which exert positive impacts on plant performance.

## Materials and methods

### Experimental design and rhizosphere soil sample collection

A series of mesocosm experiments was established in a greenhouse (average daytime temperature of 28°C (12 h), average night temperature of 25°C (12 h), and all-day average humidity of 50%) using plastic pots located at Nanjing Agriculture University (Nanjing County, Jiangsu Province, China). Soils for mesocosm experiments were collected from the chemical fertilizer application treated area of a long-term field experiment site. Detailed information of the field experiment is described in [25]. Soils were sieved through a 20 mm mesh for homogenization, with a portion (~50 kg) sterilized by gamma ray irradiation (60 KGy). The cucumber variety used in all mesocosm experiments was the commercial cultivar “Lu feng”. Each treatment in each mesocosm experiment consisted of three replicates. Each replicate contained one cucumber seedling in a plastic pot filled with 150 g of dry soil. The plastic pots were periodically randomized every 2 days throughout the experiments.

The first part of mesocosm experiment 1 was performed in nonsterilized soils to determine the effects of *C. lenta* on rhizosphere bacterial community and its links with cucumber plant biomass. The second part of mesocosm experiment 1 was performed in sterilized soils to test the direct effects of *C. lenta* on plant growth. The pure protist strain *C. lenta* ECO-P-01 used in this study was isolated and identified as previously described [26]. The detailed procedures for isolating and identifying *C. lenta* ECO-P-01 are provided in the Supplementary Methods. This protist strain was deposited in 2015 by ECOstyle BV at the Leibniz Institute DSMZ-German Collection of Microorganisms and Cell Cultures. For protistan cultures used in mesocosm experiments, the pure *C. lenta* ECO-P-01 was propagated using inactivated *Escherichia coli* DH5α (cultures were autoclaved 3 times at 121°C for 30 min) as the sole food in Page’s amoeba saline. The detailed descriptions of growth conditions and the preparation of *C. lenta* ECO-P-01 are provided in the Supplementary Methods. Four treatments were designed in mesocosm experiment 1, and a detailed description of these treatments is shown in Table S1. Mesocosm experiment 2 was established in sterilized soils to determine the predation intensity of *C. lenta* on selected bacterial strains. Given the demonstrated links between *C. lenta*, responsive bacterial taxa (*Pseudomonas*, *Sphingomonas*, and *Chitinophaga*), and plant growth improvement in the analyses of data, we isolated bacteria from the rhizosphere soil in the first mesocosm experiment and identified these isolates according to previously described protocols [27]. The detailed descriptions of the processes for isolating and identifying bacterial strains are provided in the Supplementary Methods. We selected *Pseudomonas*, *Sphingomonas*, and *Chitinophaga* strains from the rhizosphere bacterial collection for our mesocosm experiments. Twenty-two treatments were designed in mesocosm experiment 2, and a detailed description of these treatments is shown in Table S2. Mesocosm experiment 3 was established in sterilized soils to determine interactions among selected bacterial strains and links with cucumber plant biomass. Fifty treatments were designed in mesocosm experiment 3, and a detailed description of these treatments is shown in Table S3. Mesocosm experiment 4 was established in sterilized soils to determine the impacts of *C. lenta* on interactions between the plant-beneficial consortium and its antagonistic bacteria, and their links with plant growth promotion. Four treatments were designed in mesocosm experiment 4, and a detailed description of these treatments is shown in Table S4. The detailed descriptions of the processes in mesocosm experiments are provided in Supplementary Methods.

### DNA extraction and quantitative PCR assay

DNA was extracted from 0.5 g of rhizosphere soil using DNeasy PowerSoil Pro Kits (Qiagen, Germany) following the manufacturer’s instructions. DNA was stored at −20°C for quantitative PCR (qPCR) analysis and MiSeq sequencing. qPCR assays were used to quantify the abundances of 18S rRNA genes, *Pseudomonas*, *Sphingomonas*, and *Chitinophaga* using the specific primer sets (TAReuk454FWD1/TAReukREV3 [28], Ps-for/Ps-rev [29], Sphingo108f/Sphingo420r [30], and Chi forward/Chi reverse [31]) and established protocols. qPCR assays were performed on a QuantStudio 6 Flex Real-Time PCR System (Applied Biosystems, USA). The target gene copy numbers of the microorganisms are expressed as log10 values.

### MiSeq sequencing and bioinformatic analyses

The V4–5 hypervariable region of the prokaryotic 16S rRNA gene and the V4 hypervariable region of eukaryotic 18S rRNA gene were amplified with the bacterial primer set 515F/907R [32] and the eukaryote-wide primer set V4_1f/TAReukREV3 [33], respectively. Amplicons sequencing was performed on a NovaSeq PE250 (Illumina) platform at Magigene Biotech Co., Ltd. (Guangzhou, China). Sequencing libraries of bacteria and eukaryotes were constructed according to previously described protocols [34, 35]. Corresponding OTU table was obtained using the UPARSE pipeline following previously described protocols [34, 35]. The representative sequences for each bacterial and eukaryotic OTU were classified against the RDP Bacterial 16S database (v18) [36] and Protist Ribosomal Reference database (PR2) (v5.0.0) [37], respectively. A protistan OTU table was obtained according to previously described protocols [24, 34].

### Plant growth-promoting traits of selected bacterial strains

Given the observed links between the responsive bacteria (*Pseudomonas*, *Sphingomonas*, and *Chitinophaga*) and plant biomass, we selected these bacterial strains to track their plant-growth promoting traits (inorganic P solubilization, K solubilization, nitrogen fixation, ACC deaminase activity, ammonia production, IAA production, and siderophore production) in coculture and monoculture systems. The plant growth-promoting traits of selected bacterial strains in coculture and monoculture systems were tested according to previously described protocols [38–43]. Detailed processes and treatments are provided in Supplementary Methods.

### Biofilm formation of selected bacterial strains and *C. lenta* in monoculture and coculture systems

We estimated biofilm formation of selected bacterial strains and *C. lenta* in both monoculture and coculture systems. Biofilm formation was assayed and quantified according to previously described protocols [29, 44]. Detailed processes and treatments are provided in Supplementary Methods**.** In brief, the biofilms of the monoculture and coculture systems were cultivated in 200 μl inoculum (bacterial cells or protistan cells or bacterial cells + protistan cells) at 30°C for 3 days on Nunc-TSP plates. After 3 days of incubation at 30°C, we quantified biofilm formation in the monoculture and coculture systems using a modified crystal violet assay [44, 45].

### Rhizosphere soil available P and inorganic P solubilization gene category abundances determination

Rhizosphere soils from our mesocosm experiments were air-dried and sieved through a 2 mm sieve. Rhizosphere soil available P was extracted using sodium bicarbonate extraction [46, 47]. Considering the low quantity of rhizosphere soil obtained, we used 1 g rhizosphere soil samples for available P extraction. In brief, a 1 g soil sample was placed in a dry 50 ml centrifuge tube with 0.1 g of phosphorus-free activated carbon and 5 ml of sodium bicarbonate extract. The centrifuge tube was shaken vigorously on a rotary shaker (ZQZY-70B, Zhichu, China) at 180 r/min for 30 minutes. After that, the solution was obtained by filtration with non-phosphorus filter paper. The available P of the solution was measured using the molybdenum blue method according to previously described protocols [46]. The inorganic P solubilization gene category contains the pqq C and gcd genes [48]. A qPCR assay was used to quantify the copy numbers of the pqq C and gcd genes using specific primers (Table S5) according to previously established protocols [48, 49].

### Statistical analyses

The Shannon index was calculated to estimate bacterial diversity using Mothur [50]. Nonmetric multidimensional scaling based on a Bray–Curtis dissimilarity matrix was used to show rhizosphere bacterial community structures of different treatments in R software (v4.1.1). Permutational multivariate analysis of variance (PERMANOVA) was conducted to test dissimilarities of different rhizosphere bacterial communities using the “vegan” package [51] in R software (v4.1.1). Random forest models were applied to determine the main predictors of plant growth promotion via the “randomForest” package [52] in R software (v4.1.1). The importance of each metric was expressed as the percent increase in mean square error (% IncMSE) [52]. The significance of each metric was tested by the “rfPermute” package [53] in R software (v4.1.1). Multiple comparisons of the non-homogeneously distributed data were performed using Welch one-way test, followed by pairwise t-test in R software (v4.1.1). The enriched and depleted bacterial taxa (|log2Fold Change | > 1, *P* < 0.05) in the predatory protist *C. lenta* inoculation treatments were determined by the “DESeq2” package [54] in R software (v4.1.1). The *P* values were adjusted by the false discovery rate using the Benjamini-Hochberg procedure. Significant differences between different treatments were calculated by one-way ANOVA with Tukey’s HSD using SPSS software (v23). Spearman’s correlation analysis was conducted in SPSS software (v23). The formula for the predation intensity of *C. lenta* on selected bacterial strain was (Dc–Dp)/Dc, where Dc represents the selected bacterial density in the control treatment (the selected bacterial strain alone in sterilized soil), and Dp represents the selected bacterial density in the test treatment (co-inoculation of that strain and *C. lenta* in sterilized soil) [24, 55].

## Results

### Effects of *C. lenta* inoculation on the abundance of *C. lenta*, plant biomass and rhizosphere bacterial community composition

Inoculation with different densities of *C. lenta* increased the relative abundance of *C. lenta* compared to the control (no protist inoculation). We observed an increase of 874.5% in the *Cer* (10^1^) treatment, 4672.3% in the *Cer* (10^2^) treatment, and 8663.8% in the *Cer* (10^3^) treatment. These values corresponded to absolute abundance increases of 96.8% in the *Cer* (10^1^) treatment, 174.2% in the *Cer* (10^2^) treatment, and 230.1% in the *Cer* (10^3^) treatment (Tukey’s HSD test, *P* < 0.05; Fig. S1).

**Figure 1:**
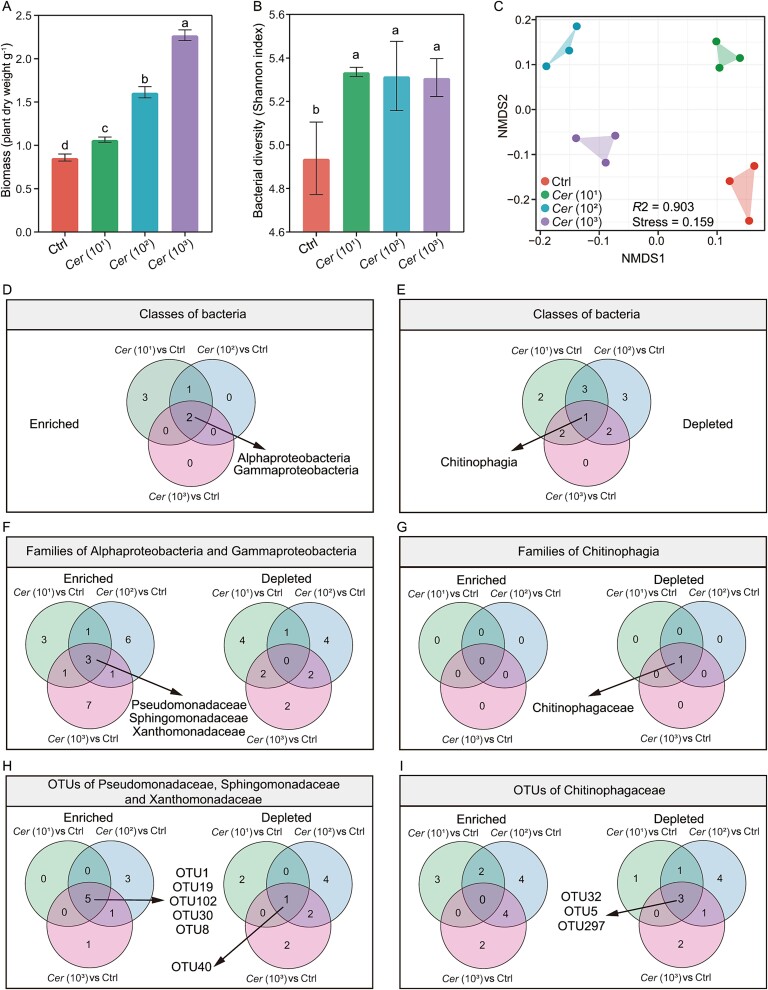
Effects of different densities of the predatory protist *C. lenta* inoculation on cucumber plant biomass (A), bacterial diversity (B), and bacterial community structure (C) in the rhizosphere. Classes of bacteria that were enriched (D) or depleted (E) in the treatments with different inoculation densities of the predatory protist *C. lenta* compared to the control. Families of Alphaproteobacteria and Gammaproteobacteria (F) and Chitinophagia (G) that were enriched or depleted in the treatments with different densities of the predatory protist *C. lenta* inoculation compared to the control. OTUs of Pseudomonadaceae, Sphingomonadaceae and Xanthomonadaceae (H), and Chitinophagaceae (I) that were enriched or depleted in different densities of the predatory protist *C. lenta* inoculation compared to the control. Ctrl = control (no protist was inoculated), *Cer* (10^1^) = *C. lenta* inoculated at 1.0 × 10^1^ cells g^−1^ dry soil, *Cer* (10^2^) = *C. lenta* inoculated at 1.0 × 10^2^ cells g^−1^ dry soil, *Cer* (10^3^) = *C. lenta* inoculated at 1.0 × 10^3^ cells g^−1^ dry soil. In panels a and B, different lowercases above the bars indicate significant differences (*P* < 0.05) between different treatments (one-way ANOVA with Tukey’s HSD test was used to calculate statistical significance).

Inoculation with different densities of *C. lenta* increased plant biomass (increase of 24.0% with *Cer* (10^1^), increase of 87.6% with *Cer* (10^2^), and increase of 164.3% with *Cer* (10^3^)) compared to the control (Tukey’s HSD test, *P* < 0.05; Fig. 1A). The highest plant biomass was observed in the *Cer* (10^3^) treatment among all *C. lenta* inoculation treatments (Tukey’s HSD test, *P* < 0.05; Fig. 1A). No significant differences in plant biomass were found among the treatments with different densities of *C. lenta* inoculation in sterilized soils (Tukey’s HSD test, *P* = 0.89; Fig. S2).

Inoculation with different densities of *C. lenta* increased the diversity of bacteria in comparison to the control in the rhizosphere (increase of 8.0% with *Cer* (10^1^), increase of 7.7% with *Cer* (10^2^), increase of 7.5% with *Cer* (10^3^); Tukey’s HSD test, *P* < 0.05; Fig. 1B). In addition, inoculation with different densities of *C. lenta* altered rhizosphere bacterial community composition compared to the control (PERMANOVA, *P* < 0.05; Fig. 1C).

We further determined the bacterial taxa that were affected by the inoculation with different densities of *C. lenta.* Among bacterial classes, Alphaproteobacteria and Gammaproteobacteria were enriched and Chitinophagia was depleted in *Cer* (10^1^), *Cer* (10^2^), and *Cer* (10^3^), in comparison to the control (DESeq2, *P* < 0.05; Fig. 1D and 1E). Among the families of the classes Alphaproteobacteria and Gammaproteobacteria, Pseudomonadaceae, Sphingomonadaceae, and Xanthomonadaceae were enriched in *Cer* (10^1^), *Cer* (10^2^), and *Cer* (10^3^), in comparison to the control (DESeq2, *P* < 0.05; Fig. 1F). Among the families of the class Chitinophagia, Chitinophagaceae was depleted in *Cer* (10^1^), *Cer* (10^2^), and *Cer* (10^3^), in comparison to the control (DESeq2, *P* < 0.05; Fig. 1G). Among the OTUs of the families Pseudomonadaceae, Sphingomonadaceae, and Xanthomonadaceae, *Pseudomonas* OTU1, *Sphingomonas* OTU19, *Sphingopyxis* OTU102, *Luteimonas* OTU30, and *Lysobacter* OTU8 were enriched, and *Arenimonas* OTU40 was depleted in *Cer* (10^1^), *Cer* (10^2^), and *Cer* (10^3^), in comparison to the control (DESeq2, *P* < 0.05; Fig. 1H). Among the OTUs of the family Chitinophagaceae, *Chitinophaga* OTU32, *Flavitalea* OTU5, and *Flavihumibacter* OTU297 were depleted in *Cer* (10^1^), *Cer* (10^2^), and *Cer* (10^3^), in comparison to the control (DESeq2, *P* < 0.05; Fig. 1I).

### Relationship between responsive rhizosphere bacterial taxa and plant biomass

**Figure 2:**
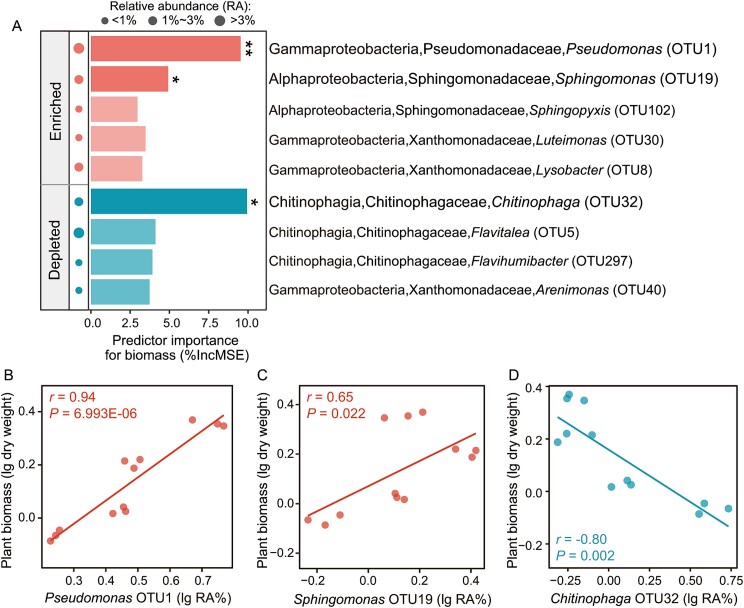
Explanatory power of responsive rhizosphere bacterial OTUs (induced by the predatory protist *C. lenta* inoculation) for cucumber plant biomass (A). Correlations between cucumber plant biomass and the relative abundances of *Pseudomonas* OTU1 (B), *Sphingomonas* OTU19 (C), and *Chitinophaga* OTU32 (D). In panel a, circles represent the average relative abundances of bacterial OTUs. * means *P* < 0.05 and ** means *P* < 0.01. In panels B, C and D, RA = relative abundance*.*

Among the responsive rhizosphere bacterial OTUs (simultaneously enriched and depleted in *C. lenta* inoculation treatments compared with the control), *Pseudomonas* OTU1 (random forest model, 9.5% increase in the mean squared error (MSE), *P* < 0.05), *Sphingomonas* OTU19 (random forest model, 4.9% increase in the MSE, *P* < 0.05) and *Chitinophaga* OTU32 (random forest model, 9.9% increase in the MSE, *P* < 0.05) were the main microbial predictors of plant biomass (Fig. 2A). The relative abundances of *Pseudomonas* OTU1 (*r* = 0.94) and *Sphingomonas* OTU19 (*r* = 0.65) were positively correlated with plant biomass (Spearman’s correlation, *P* < 0.05; Fig. 2B and 2C). The relative abundance of *Chitinophaga* OTU32 (*r* = −0.80) was negatively correlated with plant biomass (Spearman’s correlation, *P* < 0.05; Fig. 2D).

### Plant growth promoting capability of selected bacterial strains and its trophic interactions with *C. lenta*

**Figure 3:**
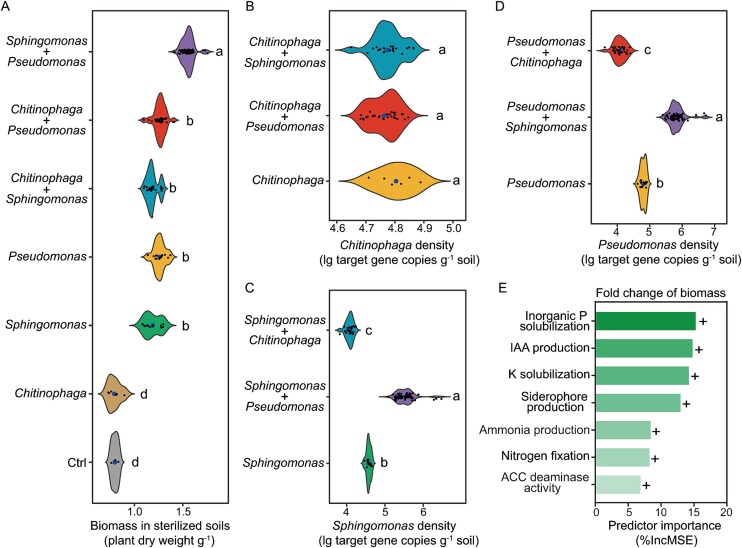
Effects of individual and mixed inoculations with selected bacterial strains on cucumber plant biomass (A) and densities of *Chitinophaga* (B), *Sphingomonas* (C), and *Pseudomonas* (D). Explanatory power of plant-growth promoting traits of selected bacterial strains for the fold change of cucumber plant biomass (E). In panels a, B, C and D, different lowercases indicate significant differences (*P* < 0.05) between different treatments (Welch one-way test with pairwise t-test was used to calculate statistical significance). Ctrl = control (no bacteria was inoculated), *Chitinophaga* = *Chitinophaga* strains were inoculated, *Sphingomonas* = *Sphingomonas* strains were inoculated, *Pseudomonas* = *Pseudomonas* strains were inoculated. In panel E, plus signs denote significant positive correlations between the plant growth-promoting traits of selected bacterial strains and the change in cucumber plant biomass. The formula for the fold change of cucumber plant biomass was (B − ctrl)/ctrl, where B is the cucumber plant biomass in different treatments with co-inoculation and separate inoculation with different selected bacterial strains, and ctrl represents the cucumber plant biomass in treatments without inoculation with selected bacterial strains.

The indigenous bacterial strains were isolated from the rhizosphere soil of above experiment. As *Pseudomonas* OTU1, *Sphingomonas* OTU19, and *Chitinophaga* OTU32 were the main microbial predictors of plant biomass in responsive rhizosphere bacterial taxa (Fig. 2), we selected *Pseudomonas*, *Sphingomonas*, and *Chitinophaga* strains from the bacterial strain collection for subsequent mesocosm experiments. Plant biomass increased after inoculation with *Pseudomonas* (increase of 58.0%) and *Sphingomonas* (increase of 48.9%; pairwise t-test, *P* < 0.05; Fig. 3A and Fig. S3), and no significant differences were found between *Chitinophaga* inoculation treatments and the control (pairwise t-test, *P* = 0.86; Fig. 3A and Fig. S3). *Pse*1 and *Sph*3 resulted in the highest plant biomass within *Pseudomonas* and *Sphingomonas* inoculation treatments, respectively (Tukey’s HSD test, *P* < 0.05; Fig. S3). Among *Pseudomonas* strains, *Pse*1 displayed the strongest ability to promote plant growth (Tukey’s HSD test, *P* < 0.05; Fig. S3) and had the highest degree of sequence identity (99.0%, 198 bp included) with *Pseudomonas* OTU1. Among *Sphingomonas* strains, *Sph*3 showed the highest promotion of plants (Tukey’s HSD test, *P* < 0.05; Fig. S3) and had the highest degree of sequence identity (99.5%, 199 bp included) with *Sphingomonas* OTU19. In addition, the predatory protist *C. lenta* had lower predation intensities on *Pseudomonas* and *Sphingomonas* than on *Chitinophaga* (Tukey’s HSD test, *P* < 0.05; Fig. S4). *C. lenta* had the lowest predation intensity on *Pse*1 among *Pseudomonas* strains and on *Sph*3 among *Sphingomonas* strains (Tukey’s HSD test, *P* < 0.05; Fig. S4).

### Interactions between selected bacterial strains and links with plant biomass

Co-inoculation with *Pseudomonas* and *Sphingomonas* increased plant biomass compared with separate *Pseudomonas* (increase of 57.9%) and *Sphingomonas* (increase of 32.8%) inoculation (pairwise t-test, *P* < 0.05; Fig. 3A and Fig. S3). No significant differences in plant biomass were found between co-inoculation with *Chitinophaga* and *Pseudomonas* and separate inoculations with *Pseudomonas* (pairwise t-test test, *P* = 0.86) or between co-inoculation with *Chitinophaga* and *Sphingomonas* and separate inoculation with *Sphingomonas* (pairwise t-test test, *P* = 0.81; Fig. 3A and Fig. S3). Co-inoculation with *Pseudomonas* and *Sphingomonas* increased plant biomass compared with co-inoculation with *Chitinophaga* and *Pseudomonas* (increase of 18.7%) and co-inoculation with *Chitinophaga* and *Sphingomonas* (increase of 31.9%; pairwise t-test, *P* < 0.05; Fig. 3A and Fig. S3). *Pse 1 + Sph3* resulted in the highest plant biomass within all co-inoculation treatments of *Pseudomonas* and *Sphingomonas* (Tukey’s HSD test, *P* < 0.05; Fig. S3).

The density of *Chitinophaga* was marginally affected by separate *Chitinophaga* inoculation, co-inoculation with *Chitinophaga* and *Pseudomonas* and co-inoculation with *Chitinophaga* and *Sphingomonas* (pairwise t-test, *P* = 0.30; Fig. 3B and Fig. S3). Co-inoculation with *Pseudomonas* and *Sphingomonas* increased the densities of *Pseudomonas* (increase of 21.3%) and *Sphingomonas* (increase of 21.0%) in comparison to separate inoculation with *Pseudomonas* and *Sphingomonas*, respectively (pairwise t-test*, P* < 0.05; Fig. 3C and 3D and Fig. S3). In contrast, co-inoculation with *Chitinophaga* and *Sphingomonas* decreased the density of *Sphingomonas* (decrease of 11.0%) in comparison to separate *Sphingomonas* inoculation (pairwise t-test, *P* < 0.05; Fig. 3C and Fig. S3). Co-inoculation with *Chitinophaga* and *Pseudomonas* decreased the density of *Pseudomonas* (decrease of 14.9%) in comparison to separate *Pseudomonas* inoculation (pairwise t-test, *P* < 0.05; Fig. 3D and Fig. S3).

We subsequently determined plant growth-promoting traits of selected bacterial strains (e.g. inorganic P solubilization, K solubilization, nitrogen fixation, ACC deaminase activity, ammonia production, IAA production, and siderophore production) in coculture and monoculture systems (Fig. S5). We found that these plant growth-promoting traits of the selected bacterial strains were positively correlated with the change in plant biomass in the coculture and monoculture systems (Spearman’s correlation, *P* < 0.05; Fig. 3E and Fig. S5). In particular, the inorganic P solubilization ability was the greatest predictor of the increase in plant biomass (random forest model, 15.9% increase in the MSE; Fig. 3E).

### Effects of *C. lenta* on the biofilm formation of selected bacterial strains

**Figure 4:**
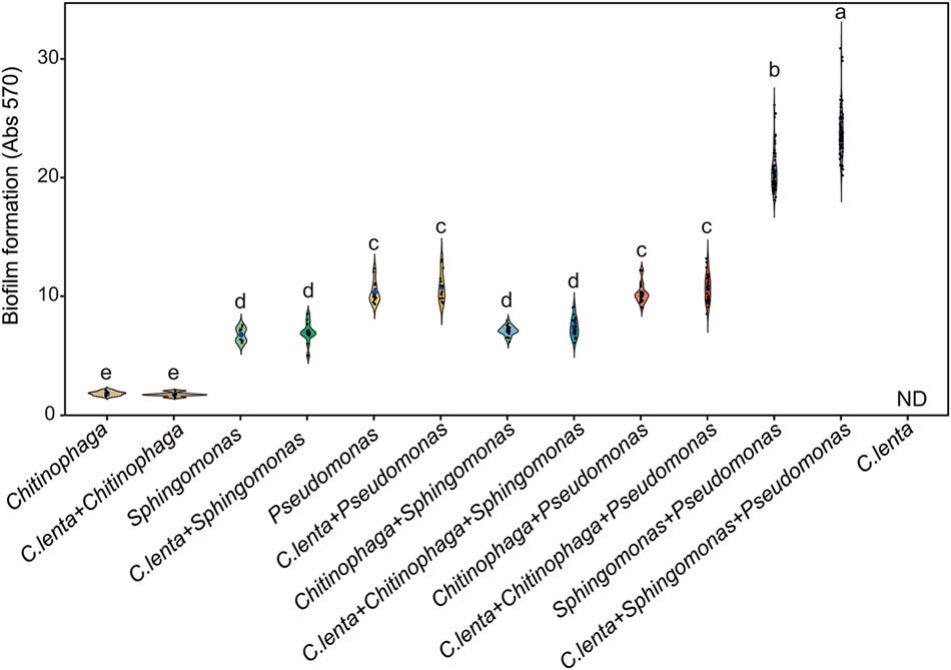
Biofilm formation of selected bacterial strains and the predatory protist *C. lenta* in coculture and monoculture systems. Different lowercases indicate significant differences (*P* < 0.05) between different treatments (Welch one-way test with pairwise t-test was used to calculate statistical significance). ND = no value was detected.

We further evaluated the biofilm formation of selected bacterial strains and *C. lenta* in coculture and monoculture systems. In culture systems of selected bacterial strains, we found that the biofilm formation of individual cultures of *Pseudomonas* (increase of 459.7%) and *Sphingomonas* (increase of 52.5%) was greater than that of individual cultures of *Chitinophaga* (pairwise t-test, *P* < 0.05; Fig. 4 and Fig. S6). Biofilm formation in coculture systems combining *Pseudomonas* with *Sphingomonas* was higher than of the respective monocultures (increase of 99.6% for *Pseudomonas* monoculture and increase of 204.4% for *Sphingomonas* monoculture; pairwise t-test, *P* < 0.05; Fig. 4 and Fig. S6). No significant differences in biofilm formation were found between the coculture of *Pseudomonas* and *Chitinophaga* and the monoculture of *Pseudomonas* (pairwise t-test, *P* = 0.68) or between the coculture of *Sphingomonas* and *Chitinophaga* and the monoculture of *Sphingomonas* (pairwise t-test, *P* = 0.13; Fig. 4 and Fig. S6). Biofilm formation in coculture systems combining *Pseudomonas* with *Sphingomonas* was greater than that of *Pseudomonas* with *Chitinophaga* (increase of 100.6%) and *Sphingomonas* with *Chitinophaga* (increase of 190.1%; pairwise t-test, *P* < 0.05; Fig. 4 and Fig. S6). *Pse 1 + Sph3* had the greatest biofilm formation in coculture systems combining *Pseudomonas* with *Sphingomonas* (Tukey’s HSD test, *P* < 0.05; Fig. S6).

In culture systems combining selected bacterial strains with *C. lenta*, we found that biofilm formation in coculture systems of *Pseudomonas*, *Sphingomonas*, and *C. lenta* was greater than that of *Pseudomonas* and *Sphingomonas* (increase of 15.8%; pairwise t-test, *P* < 0.05; Fig. 4 and Fig. S6). No significant differences in biofilm formation were found between the coculture of *C. lenta* and *Chitinophaga* and the monoculture of *Chitinophaga* (pairwise t-test, *P* = 0.44), between the coculture of *C. lenta* and *Sphingomonas* and the monoculture of *Sphingomonas* (pairwise t-test, *P* = 0.54), between the coculture of *C. lenta* and *Pseudomonas* and the monoculture of *Pseudomonas* (pairwise t-test, *P* = 0.25), between the coculture of *C. lenta*, *Chitinophaga*, and *Sphingomonas* and the coculture of *Chitinophaga* and *Sphingomonas* (pairwise t-test, *P* = 0.12) or between the coculture of *C. lenta*, *Chitinophaga*, and *Pseudomonas* and the coculture of *Chitinophaga* and *Pseudomonas* (pairwise t-test, *P* = 0.10; Fig. 4 and Fig. S6).

### Effects of *C. lenta* on interactions between the plant-beneficial consortium and its antagonists and links with plant biomass

As *Pseudomonas* strain 1 and *Sphingomonas* strain 3 had the greatest plant growth-promoting and inorganic P solubilization ability and had the highest degree of sequence identity with responsive bacterial OTUs, we used *Pseudomonas* strain 1(*Pse*1), *Sphingomonas* strain 3 (*Sph*3), and *Chitinophaga* strain 1 (*Chi*1) combined with *C. lenta* for subsequent experiments. In the subsequent microcosm assay, we explored the effects of *C. lenta* on interactions between the simple growth-promoting rhizobacterial consortium (*Pseudomonas* strain 1*+ Sphingomonas* strain 3) and its antagonistic bacteria (*Chitinophaga* strain 1). We further examined the impact of bacterial interactions mediated by *C. lenta* predation on rhizosphere inorganic P solubilization enhancement and links with plant growth improvement.

**Figure 5:**
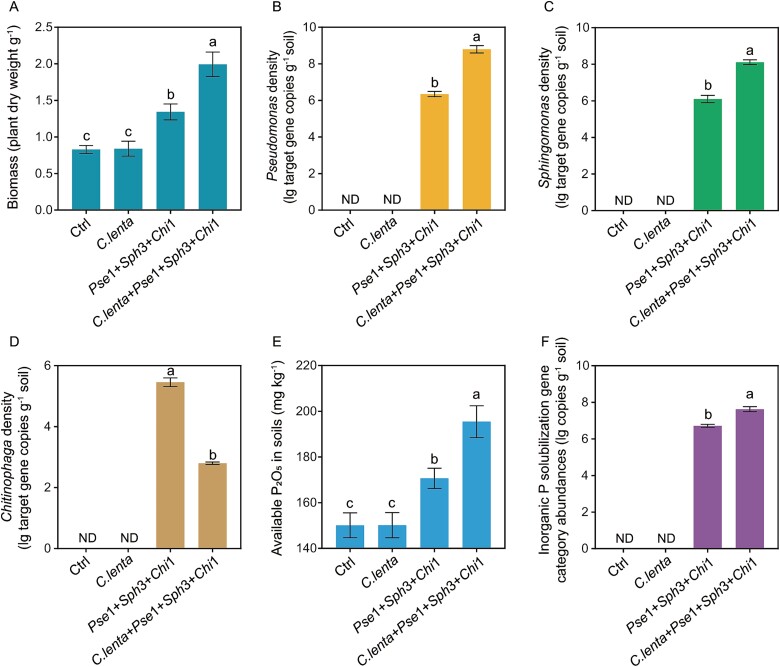
Cucumber plant biomass (A), densities of *Chitinophaga* (B), *Sphingomonas* (C), and *Pseudomonas* (D), available P_2_O_5_ contents (E), and inorganic P solubilization gene category abundances (F) in treatments with individual and mixed inoculation with *Pseudomonas* strain 1, *Sphingomonas* strain 3, *Chitinophaga* strain 1, and the predatory protist *C. lenta* in sterilized soils. Different lowercases above the bars indicate significant differences (*P* < 0.05) between different treatments (one-way ANOVA with Tukey’s HSD test was used to calculate statistical significance). Ctrl = control (no microbe was inoculated), *chi* 1 = *Chitinophaga* strain 1, *Sph* 3 = *Sphingomonas* strain 3, *pes* 1 = *Pseudomonas* strain 1, ND = no value was detected.

*Pse*1 + *Sph*3 + *Chi*1 and *C. lenta + Pse*1 + *Sph*3 + *Chi*1 had the higher plant biomass compared with Ctrl and *C. lenta* (Tukey’s HSD test, *P* < 0.05), and no significant differences were found between Ctrl and *C. lenta* (Tukey’s HSD test, *P* = 0.89; Fig. 5A). *C. lenta + Pse*1 + *Sph*3 + *Chi*1 had the higher plant biomass (increase of 48.6%) and the densities of *Pseudomonas* (increase of 38.6%) and *Sphingomonas* (increase of 33.0%) in comparison to *Pse*1 + *Sph*3 + *Chi*1 (Tukey’s HSD test, *P* < 0.05; Fig. 5A, 5B, and 5C). In contrast*, C. lenta + Pse*1 + *Sph*3 + *Chi*1 had the lower density of *Chitinophaga* (decrease of 48.7%) compared with *Pse*1 + *Sph*3 + *Chi*1 (Tukey’s HSD test, *P* < 0.05; Fig. 5D)*.* In addition, *Pse*1 + *Sph*3 + *Chi*1 and *C. lenta + Pse*1 + *Sph*3 + *Chi*1 had the higher available P_2_O_5_ contents compared with Ctrl and *C. lenta* (Tukey’s HSD test, *P* < 0.05; Fig. 5E), and no significant differences were found between Ctrl and *C. lenta* (Tukey’s HSD test, *P* = 0.99; Fig. 5E). *C. lenta + Pse*1 + *Sph*3 + *Chi*1 had the higher available P_2_O_5_ contents (increase of 14.5%) and inorganic P solubilization gene category abundances (increase of 13.5%) in comparison to *Pse*1 + *Sph*3 + *Chi*1 (Tukey’s HSD test, *P* < 0.05; Fig. 5E and 5F).

## Discussion

In this study, we sought to gain insight into the mechanisms by which protists contribute to improved plant performance. We specifically zoomed in on the effects of protist inoculation on bacterial communities in the rhizosphere, interactions between different protist-effected bacterial taxa, and the potential impacts of such interactions on rhizosphere colonization and plant growth promotion. We found that the predatory protist *C. lenta* enriched for specific microbial taxa (*Pseudomonas* and *Sphingomonas*), whose interactions lead to improved biofilm formation, rhizosphere colonization, and P mobilization, and ultimately improved plant performance.

Our data support our first hypothesis that *C. lenta* would impact the rhizosphere bacterial community so as to enrich specific plant-beneficial bacteria, thereby enhancing plant performance. We found that the application of *C. lenta* induced changes in the rhizosphere bacterial community that could be linked to improvements in plant performance. This suggests that *C. lenta* is an important top-down regulator of rhizosphere microbiome community composition [16, 56]. We further identified specific bacterial taxa, such as *Pseudomonas* and *Sphingomonas* that were impacted by *C. lenta* inoculation and determined their explanatory link with plant performance. Numerous previous studies have demonstrated that *Pseudomonas* and *Sphingomonas* can act as PGPR to promote plant growth via a range of mechanisms, such as by solubilizing phosphorus and synthesizing IAA [57–60]. Our results suggest that *C. lenta* may enhance the fitness of plant-beneficial bacteria (e.g. *Pseudomonas* and *Sphingomonas*) by preying on other bacteria (e.g. *Chitinophaga*) that may inhibit the growth of plant-beneficial bacteria [61]. Previous studies have suggested that biofilm formation may represent an important mechanism by which bacteria can avoid protist predation [62, 63]. It may therefore be that protist predation at least partially selects for traits related to biofilm formation, including multi-species biofilms [64], thereby generally increasing the proportion of biofilm-producing strains that can potentially colonize the rhizosphere and impact plant performance. We indeed found that *Pseudomonas* and *Sphingomonas* had stronger biofilm formation abilities than their potential antagonist *Chitinophaga.* Therefore, *C. lenta* predation-induced shifts in the bacterial community might increase the number and performance of PGPR (e.g. *Pseudomonas* and *Sphingomonas*) in the rhizosphere, leading to enhanced plant performance.

We also found support for our second hypothesis that *C. lenta* would support cooperation within plant growth-promoting rhizobacterial consortia to elicit activities in support of plant performance. Inoculation with *C. lenta* induced the formation of a plant growth-promoting rhizobacterial consortium composed of *Pseudomonas* and *Sphingomonas*, leading to increased biofilm production. This enhanced the rhizosphere colonization of the plant growth-promoting rhizobacterial consortium, which lead to improved phosphate solubilization. Numerous previous studies have demonstrated that bacterial biofilm formation influences the colonization of bacteria in the rhizosphere of host plants [12, 13]. Moreover, bacterial biofilm formation is often linked to improved plant performance, for instance due to better competitive exclusion of pathogens [29, 65]. In addition, the phosphate solubilization provided by plant-associated bacteria is closely related to the improvement of plant performance [66]. Previous studies have also shown positive impacts of protists on plant performance, with links to the changes in the relative abundance of distinct bacterial groups in relation to protistan predation [20, 25, 67]. Here, we show that the predation of *C. lenta* induces the form of a plant growth-promoting rhizobacterial consortium that includes microbial cooperative interactions. These microbial cooperative interactions facilitate increased biofilm production and rhizosphere colonization, which improves phosphorous mobilization, eventually leading to enhanced plant performance. Our results not only further highlight the importance of plant growth-promoting rhizobacterial consortia in plant performance improvement [68], but also indicate that the role of protists in stimulating such activities should be considered in future investigations to develop new agricultural biological agents.

## Conclusion

**Figure 6:**
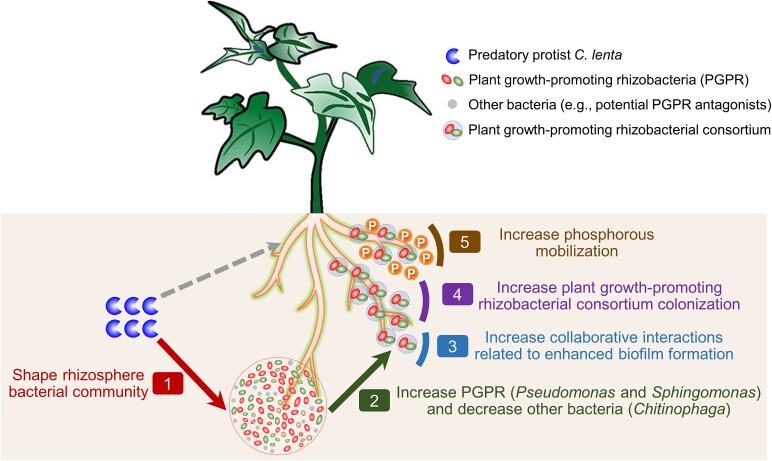
Conceptual model depicting the mechanisms underlying the interconnections in the protist–bacteria–plant triangle.

We have summarized the mechanisms underlying the interconnections within the protist–bacteria–plant triangle of interactions in a conceptual model, as depicted in Fig. 6, where *C. lenta* improves plant performance by enhancing interactions with and within the rhizosphere microbiome. Taken together, our results demonstrate that the predatory protist *C. lenta* impacts the rhizosphere bacterial community leading to more collaborative interactions related to enhanced biofilm formation, increasing the colonization of plant growth-promoting rhizobacterial consortia (*Pseudomonas* and *Sphingomonas*), leading to improved phosphorous mobilization that benefits plant performance. Therefore, we propose that protists represent attractive agents for future, microbe-based sustainable agricultural practices, both as bioagents or by targeted stimulation of resident protist populations.

## Supplementary Material

Supplementary_information3_wrae180

## Data Availability

Sequence data are available at the NCBI Sequence Read Archive (PRJNA1027582).
