# Supplementary material for: Predatory protists impact plant performance by promoting plant growth-promoting rhizobacterial consortia
Source: ISME J. 2024 Sep 23;18(1):wrae180. doi: 10.1093/ismejo/wrae180 (PMC11459550; doi:10.1093/ismejo/wrae180)
Supplement: Supplementary_information3_wrae180 [file supplementary_information3_wrae180.docx]

**Supplementary information**

**Title: Predatory protists impact plant** **performance by promoting** **plant growth-promoting rhizobacterial consortia**

Sai Guo^1^, Stefan Geisen^2^, Yani Mo^1^, Xinyue Yan^1^, Ruoling Huang^1^, Hongjun Liu^1^, Zhilei Gao^3,4^, Chengyuan Tao^1^, Xuhui Deng^1^, Wu Xiong^1^, Qirong Shen^1^, George A. Kowalchuk^4^ and Rong Li^1*^

**Running title:** **Protist–bacteria–plant interactions**

**Affiliations**

^1^ The Sanya Institute of the Nanjing Agricultural University, Jiangsu Provincial Key Lab of Solid Organic Waste Utilization, Jiangsu Collaborative Innovation Center of Solid Organic Wastes, Educational Ministry Engineering Center of Resource-saving fertilizers, Nanjing Agricultural University, Nanjing 210095, Jiangsu, Peoples R China

^2^ Laboratory of Nematology, Wageningen University, 6700 AA Wageningen, The Netherlands

^3^ EUROstyle BV, Ecomunitypark 1, 8431 SM Oosterwolde, The Netherlands

^4^ Ecology and Biodiversity Group, Department of Biology, Institute of Environmental Biology, Utrecht University, Padualaan 8, 3584 CH Utrecht, The Netherlands

^*^Corresponding author: Rong Li, College of Resources and Environmental Sciences, Nanjing Agricultural University, 210095, Nanjing, China. E-mail: lirong@njau.edu.cn. Tel: (86)02584396286. Fax: (86)02584396286.

**Supplementary figures**

**Fig. S1. Effects of different densities of the predatory protist *C. lenta* inoculation on the absolute abundance of the protist 18S rRNA genes (A), relative abundance of *C. lenta* (B), and absolute abundance of *C. lenta*(C) in nonsterilized soils.**


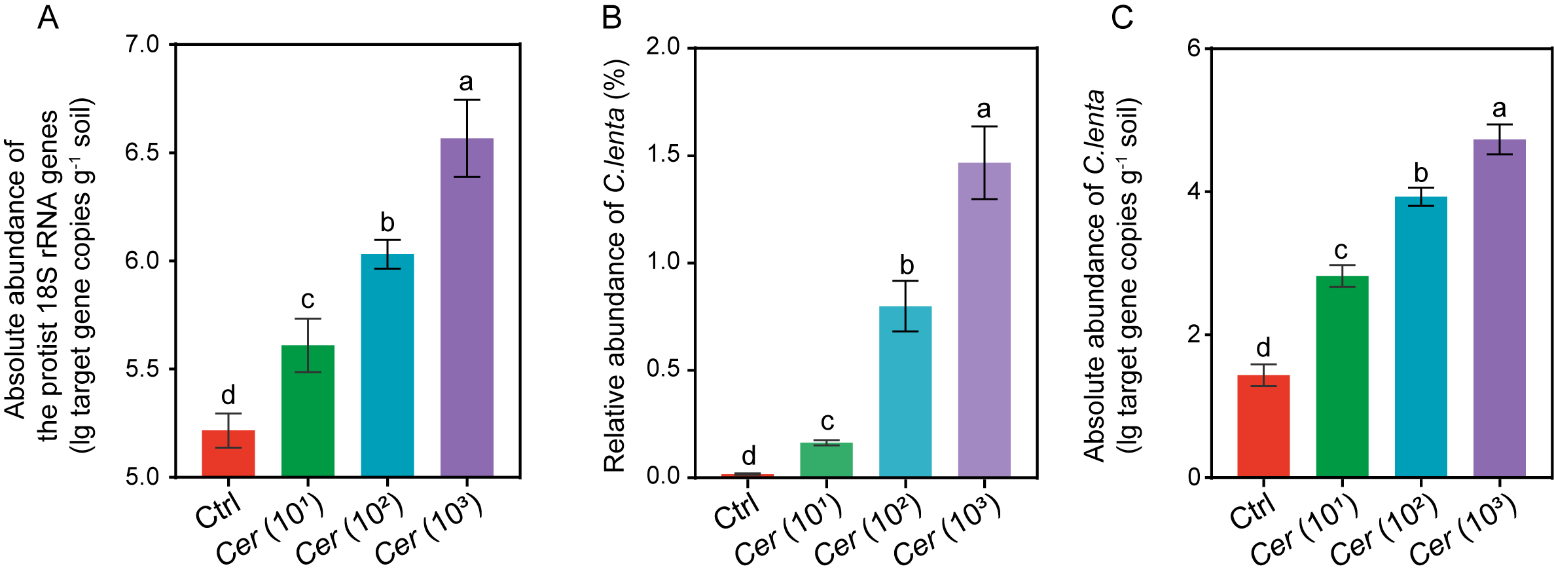


*Ctrl* = control (no protist was inoculated), *Cer* (10^1^) = *C. lenta* inoculated at 1.0×10^1^ cells g^-1^ dry soil, *Cer* (10^2^) = *C. lenta* inoculated at 1.0×10^2^ cells g^-1^ dry soil, *Cer* (10^3^) = *C. lenta* inoculated at 1.0×10^3^ cells g^-1^ dry soil. Bars with different letters indicate significant differences between different treatments as defined by one-way ANOVA with Tukey's HSD test (*P* < 0.05).

**Fig. S2. Effects of different densities of the predatory protist *C. lenta* inoculation on cucumber plant biomass in sterilized soils.**


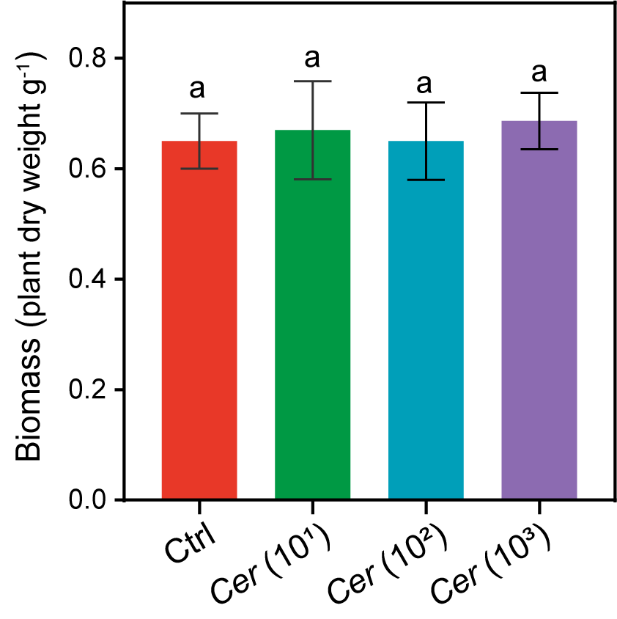


*Ctrl* = control (no protist was inoculated), *Cer* (10^1^) = *C. lenta* inoculated at 1.0×10^1^ cells g^-1^ dry soil, *Cer* (10^2^) = *C. lenta* inoculated at 1.0×10^2^ cells g^-1^ dry soil, *Cer* (10^3^) = *C. lenta* inoculated at 1.0×10^3^ cells g^-1^ dry soil. Bars with same letters indicate no significant differences between different treatments as defined by one-way ANOVA with Tukey's HSD test (*P* > 0.05).

**Fig. S3. Effects of individual and mixed inoculation with selected bacterial strains on cucumber plant biomass (A) and densities of *Chitinophaga* (B), *Sphingomonas* (C), and *Pseudomonas* (D) in sterilized soils.**


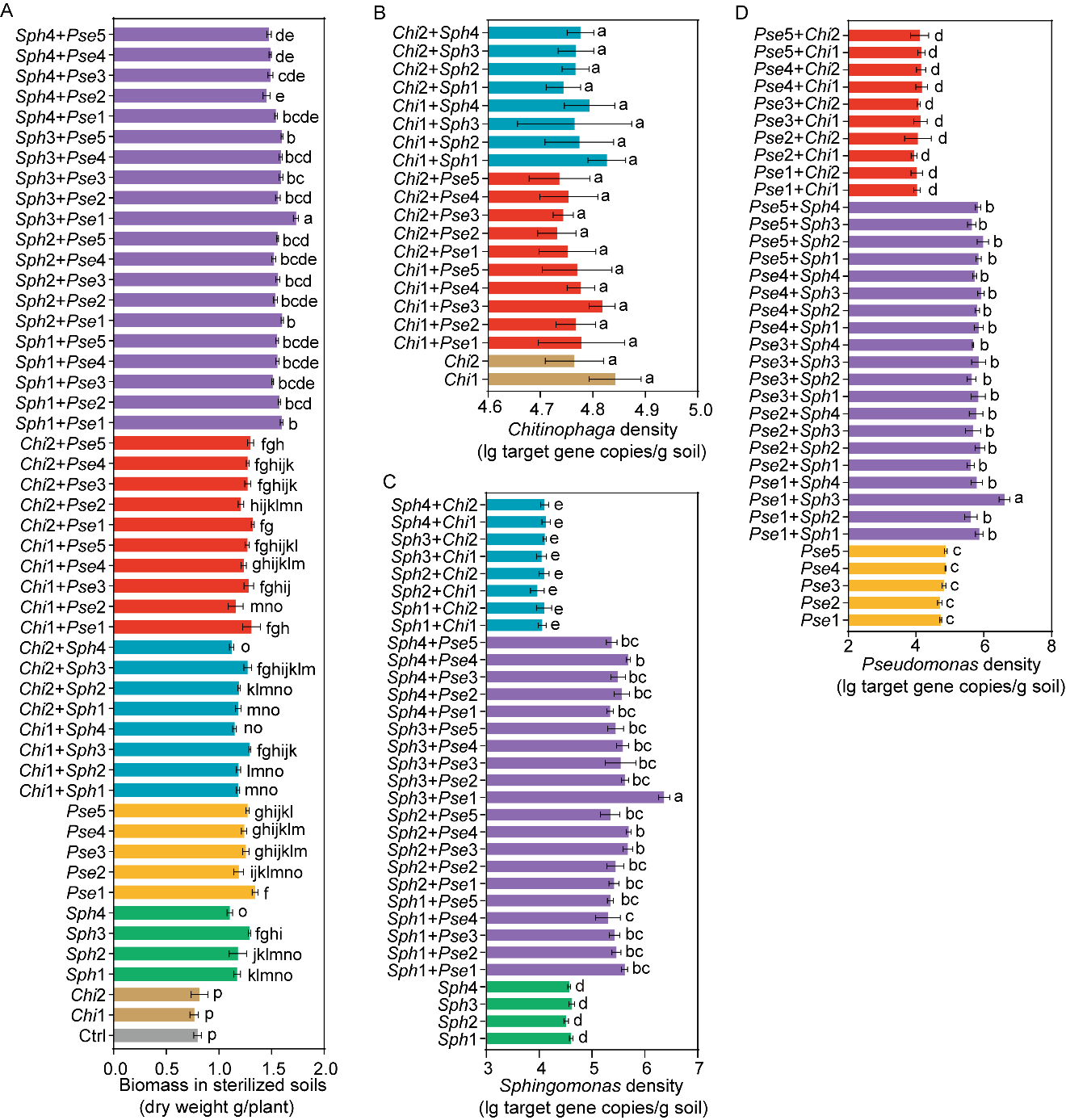


*Ctrl* = control (no bacteria was inoculated), *Chi* = *Chitinophaga* strains were inoculated, *Sph* = *Sphingomonas* strains were inoculated, *Pse*= *Pseudomonas* strains were inoculated. Bars with different letters indicate significant differences between different treatments as defined by one-way ANOVA with Tukey's HSD test (*P* < 0.05).

**Fig. S4. Predation intensity of the predatory protist *C. lenta* on selected bacterial strains.**


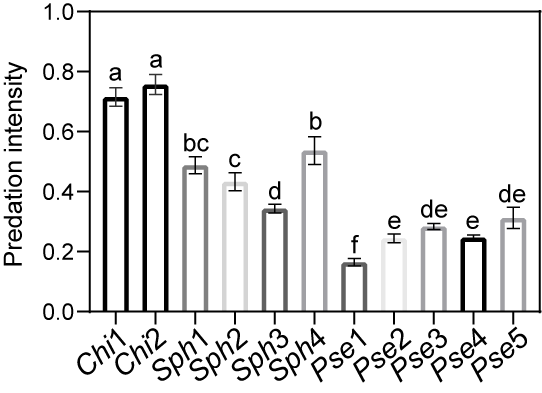


Bars with different letters indicate significant differences as defined by Tukey's HSD test (*P* < 0.05). *Chi* = *Chitinophaga* strains, *Sph* = *Sphingomona* strains, *Pes* = *Pseudomonas* strains*.*

**Fig. S5. Inorganic P solubilization (A), K solubilization (B), IAA production (C), siderophore production (D), N_2_ fixation (E), ammonia production (F), and ACC deaminase activity (G) of selected bacterial strains in coculture and monoculture systems.**


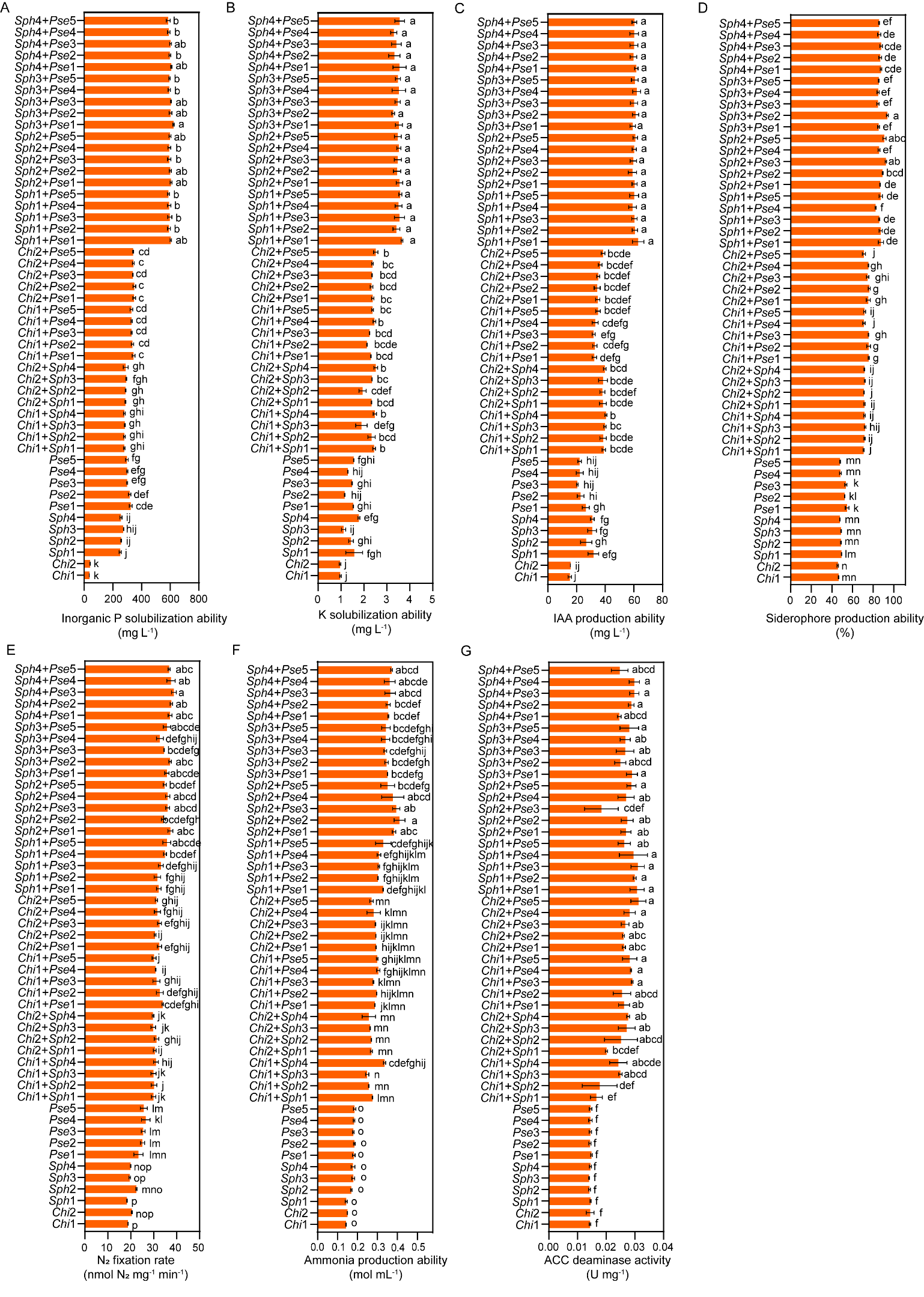


*Chi* = *Chitinophaga* strains, *Sph* = *Sphingomona* strains, *Pes* = *Pseudomonas* strains*.* Bars with different letters indicate significant differences between different treatments as defined by one-way ANOVA with Tukey's HSD test (*P* < 0.05).

**Fig. S6. Biofilm formation of selected bacterial strains and the predatory protist *C. lenta* in coculture and monoculture systems.**


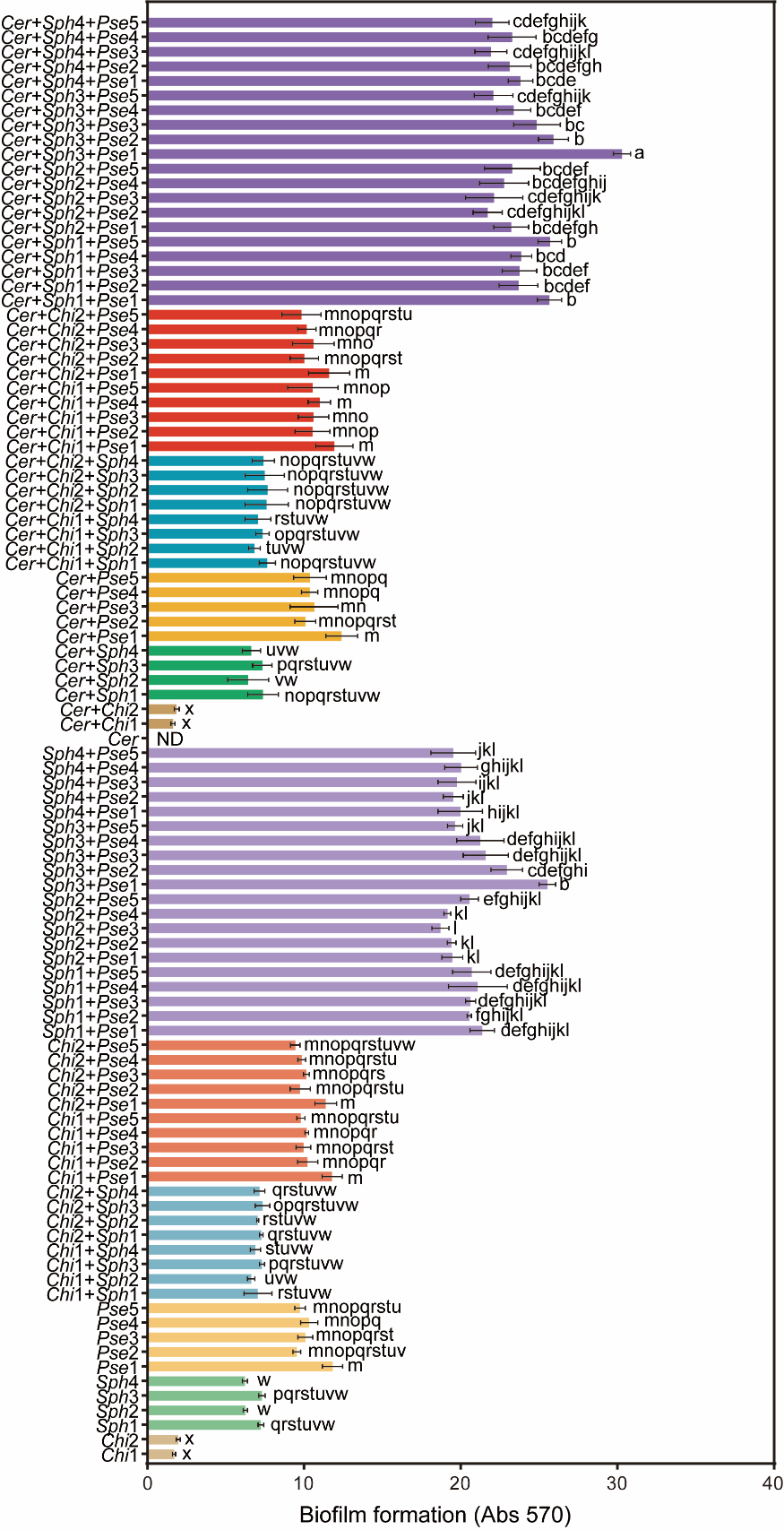


*Chi* = *Chitinophaga* strains, *Sph* = *Sphingomonas* strains, *Pse* = *Pseudomonas* strains, *Cer* = *C. lenta.* Bars with different letters indicate significant differences between different treatments as defined by one-way ANOVA with Tukey's HSD test (*P* < 0.05).

**Supplementary tables**

**Table S1. Detailed inoculation treatments of mesocosm experiment 1.**

| Treatment name | Inoculum |
| --- | --- |
| Ctrl | No protist |
| *Cer* (10^1^) | *Cercomonas lenta* strain ECO-P-01 (1.0×10^1^ cells g^-1^ dry soil) |
| *Cer* (10^2^) | *Cercomonas lenta* strain ECO-P-01 (1.0×10^2^ cells g^-1^ dry soil) |
| *Cer* (10^3^) | *Cercomonas lenta* strain ECO-P-01 (1.0×10^3^ cells g^-1^ dry soil) |

**Table S2. Detailed inoculation treatments of mesocosm experiment 2.**

| Treatment name | Inoculum |
| --- | --- |
| *Pse*1 | *Pseudomonas* strain 1 (1.0×10^4^ cells g^-1^ dry soil) |
| *Pse*1*+Cer* | *Pseudomonas* strain 1 (1.0×10^4^ cells g^-1^ dry soil) and *Cercomonas lenta* strain ECO-P-01 (1.0×10^2^ cells g^-1^ dry soil) |
| *Pse*2 | *Pseudomonas* strain 2 (1.0×10^4^ cells g^-1^ dry soil) |
| *Pse*2*+Cer* | *Pseudomonas* strain 2 (1.0×10^4^ cells g^-1^ dry soil) and *Cercomonas lenta* strain ECO-P-01 (1.0×10^2^ cells g^-1^ dry soil) |
| *Pse*3 | *Pseudomonas* strain 3 (1.0×10^4^ cells g^-1^ dry soil) |
| *Pse*3*+Cer* | *Pseudomonas* strain 3 (1.0×10^4^ cells g^-1^ dry soil) and *Cercomonas lenta* strain ECO-P-01 (1.0×10^2^ cells g^-1^ dry soil) |
| *Pse*4 | *Pseudomonas* strain 4 (1.0×10^4^ cells g^-1^ dry soil) i |
| *Pse*4*+Cer* | *Pseudomonas* strain 4 (1.0×10^4^ cells g^-1^ dry soil) and *Cercomonas lenta* strain ECO-P-01 (1.0×10^2^ cells g^-1^ dry soil) |
| *Pse*5 | *Pseudomonas* strain 5 (1.0×10^4^ cells g^-1^ dry soil) |
| *Pse*5*+Cer* | *Pseudomonas* strain 5 (1.0×10^4^ cells g^-1^ dry soil) and *Cercomonas lenta* strain ECO-P-01 (1.0×10^2^ cells g^-1^ dry soil) |
| *Sph*1 | *Sphingomonas* strain 1 (1.0×10^4^ cells g^-1^ dry soil) |
| *Sph*1*+Cer* | *Sphingomonas* strain 1 (1.0×10^4^ cells g^-1^ dry soil) and *Cercomonas lenta* strain ECO-P-01 (1.0×10^2^ cells g^-1^ dry soil) |
| *Sph*2 | *Sphingomonas* strain 2 (1.0×10^4^ cells g^-1^ dry soil) |
| *Sph*2*+Cer* | *Sphingomonas* strain 2 (1.0×10^4^ cells g^-1^ dry soil) and *Cercomonas lenta* strain ECO-P-01 (1.0×10^2^ cells g^-1^ dry soil) |
| *Sph*3 | *Sphingomonas* strain 3 (1.0×10^4^ cells g^-1^ dry soil) |
| *Sph*3*+Cer* | *Sphingomonas* strain 3 (1.0×10^4^ cells g^-1^ dry soil) and *Cercomonas lenta* strain ECO-P-01 (1.0×10^2^ cells g^-1^ dry soil) |
| *Sph*4 | *Sphingomonas* strain 4 (1.0×10^4^ cells g^-1^ dry soil) |
| *Sph*4*+* *Cer* | *Sphingomonas* strain 4 (1.0×10^4^ cells g^-1^ dry soil) and *Cercomonas lenta* strain ECO-P-01 (1.0×10^2^ cells g^-1^ dry soil) |
| *Chi*1 | *Chitinophaga* strain 1 (1.0×10^4^ cells g^-1^ dry soil) |
| *Chi*1*+Cer* | *Chitinophaga* strain 1 (1.0×10^4^ cells g^-1^ dry soil) and *Cercomonas lenta* strain ECO-P-01 (1.0×10^2^ cells g^-1^ dry soil) |
| *Chi*2 | *Chitinophaga* strain 2 (1.0×10^4^ cells g^-1^ dry soil) |
| *Chi*2*+Cer* | *Chitinophaga* strain 1 (1.0×10^4^ cells g^-1^ dry soil) and *Cercomonas lenta* strain ECO-P-01 (1.0×10^2^ cells g^-1^ dry soil) |

**Table S3. Detailed inoculation treatments of mesocosm experiment 3.**

| Treatment name | Inoculum |
| --- | --- |
| Ctrl | No bacteria |
| *Chi*1 | *Chitinophaga* strain 1 (1.0×10^4^ cells g^-1^ dry soil) |
| *Chi*2 | *Chitinophaga* strain 2 (1.0×10^4^ cells g^-1^ dry soil) |
| *Sph*1 | *Sphingomonas* strain 1 (1.0×10^4^ cells g^-1^ dry soil) |
| *Sph*2 | *Sphingomonas* strain 2 (1.0×10^4^ cells g^-1^ dry soil) |
| *Sph*3 | *Sphingomonas* strain 3 (1.0×10^4^ cells g^-1^ dry soil) |
| *Sph*4 | *Sphingomonas* strain 4 (1.0×10^4^ cells g^-1^ dry soil) |
| *Pse*1 | *Pseudomonas* strain 1 (1.0×10^4^ cells g^-1^ dry soil) |
| *Pse*2 | *Pseudomonas* strain 2 (1.0×10^4^ cells g^-1^ dry soil) |
| *Pse*3 | *Pseudomonas* strain 3 (1.0×10^4^ cells g^-1^ dry soil) |
| *Pse*4 | *Pseudomonas* strain 4 (1.0×10^4^ cells g^-1^ dry soil) |
| *Pse*5 | *Pseudomonas* strain 5 (1.0×10^4^ cells g^-1^ dry soil) |
| *Chi*1*+* *Sph*1 | *Chitinophaga* strain 1 (1.0×10^4^ cells g^-1^ dry soil) and *Sphingomonas* strain 1 (1.0×10^4^ cells g^-1^ dry soil) |
| *Chi*1*+Sph*2 | *Chitinophaga* strain 1 (1.0×10^4^ cells g^-1^ dry soil) and *Sphingomonas* strain 2 (1.0×10^4^ cells g^-1^ dry soil) |
| *Chi*1*+Sph*3 | *Chitinophaga* strain 1 (1.0×10^4^ cells g^-1^ dry soil) and *Sphingomonas* strain 3 (1.0×10^4^ cells g^-1^ dry soil) |
| *Chi*1*+Sph*4 | *Chitinophaga* strain 1 (1.0×10^4^ cells g^-1^ dry soil) and *Sphingomonas* strain 4 (1.0×10^4^ cells g^-1^ dry soil) |
| *Chi*2*+Sph*1 | *Chitinophaga* strain 2 (1.0×10^4^ cells g^-1^ dry soil) and *Sphingomonas* strain 1 (1.0×10^4^ cells g^-1^ dry soil) |
| *Chi*2*+Sph*2 | *Chitinophaga* strain 2 (1.0×10^4^ cells g^-1^ dry soil) and *Sphingomonas* strain 2 (1.0×10^4^ cells g^-1^ dry soil) |
| *Chi*2*+* *Sph*3 | *Chitinophaga* strain 2 (1.0×10^4^ cells g^-1^ dry soil) and *Sphingomonas* strain 3 (1.0×10^4^ cells g^-1^ dry soil) |
| *Chi*2*+Sph*4 | *Chitinophaga* strain 2 (1.0×10^4^ cells g^-1^ dry soil) and *Sphingomonas* strain 4 (1.0×10^4^ cells g^-1^ dry soil) |
| *Chi*1*+Pse*1 | *Chitinophaga* strain 1 (1.0×10^4^ cells g^-1^ dry soil) and *Pseudomonas* strain 1 (1.0×10^4^ cells g^-1^ dry soil) |
| *Chi*1*+Pse*2 | *Chitinophaga* strain 1 (1.0×10^4^ cells g^-1^ dry soil) and *Pseudomonas* strain 2 (1.0×10^4^ cells g^-1^ dry soil) |
| *Chi*1*+Pse*3 | *Chitinophaga* strain 1 (1.0×10^4^ cells g^-1^ dry soil) and *Pseudomonas* strain 3 (1.0×10^4^ cells g^-1^ dry soil) |
| *Chi*1*+Pse*4 | *Chitinophaga* strain 1 (1.0×10^4^ cells g^-1^ dry soil) and *Pseudomonas* strain 4 (1.0×10^4^ cells g^-1^ dry soil) |
| *Chi*1*+Pse*5 | *Chitinophaga* strain 1 (1.0×10^4^ cells g^-1^ dry soil) and *Pseudomonas* strain 5 (1.0×10^4^ cells g^-1^ dry soil) |
| *Chi*2*+Pse*1 | *Chitinophaga* strain 2 (1.0×10^4^ cells g^-1^ dry soil) and *Pseudomonas* strain 1 (1.0×10^4^ cells g^-1^ dry soil) |
| *Chi*2*+Pse*2 | *Chitinophaga* strain 2 (1.0×10^4^ cells g^-1^ dry soil) and *Pseudomonas* strain 2 (1.0×10^4^ cells g^-1^ dry soil) |
| *Chi*2*+Pse*3 | *Chitinophaga* strain 2 (1.0×10^4^ cells g^-1^ dry soil) and *Pseudomonas* strain 3 (1.0×10^4^ cells g^-1^ dry soil) |
| *Chi*2*+Pse*4 | *Chitinophaga* strain 2 (1.0×10^4^ cells g^-1^ dry soil) and *Pseudomonas* strain 4 (1.0×10^4^ cells g^-1^ dry soil) |
| *Chi*2*+Pse*5 | *Chitinophaga* strain 2 (1.0×10^4^ cells g^-1^ dry soil) and *Pseudomonas* strain 5 (1.0×10^4^ cells g^-1^ dry soil) |
| *Sph*1*+Pse*1 | *Sphingomonas* strain 1 (1.0×10^4^ cells g^-1^ dry soil) and *Pseudomonas* strain 1 (1.0×10^4^ cells g^-1^ dry soil) |
| *Sph*1*+Pse*2 | *Sphingomonas* strain 1 (1.0×10^4^ cells g^-1^ dry soil) and *Pseudomonas* strain 2 (1.0×10^4^ cells g^-1^ dry soil) |
| *Sph*1*+Pse*3 | *Sphingomonas* strain 1 (1.0×10^4^ cells g^-1^ dry soil) and *Pseudomonas* strain 3 (1.0×10^4^ cells g^-1^ dry soil) |
| *Sph*1*+Pse*4 | *Sphingomonas* strain 1 (1.0×10^4^ cells g^-1^ dry soil) and *Pseudomonas* strain 4 (1.0×10^4^ cells g^-1^ dry soil) |
| *Sph*1*+Pse*5 | *Sphingomonas* strain 1 (1.0×10^4^ cells g^-1^ dry soil) and *Pseudomonas* strain 5 (1.0×10^4^ cells g^-1^ dry soil) |
| *Sph*2*+Pse*1 | *Sphingomonas* strain 2 (1.0×10^4^ cells g^-1^ dry soil) and *Pseudomonas* strain 1 (1.0×10^4^ cells g^-1^ dry soil) |
| *Sph*2*+Pse*2 | *Sphingomonas* strain 2 (1.0×10^4^ cells g^-1^ dry soil) and *Pseudomonas* strain 2 (1.0×10^4^ cells g^-1^ dry soil) |
| *Sph*2*+Pse*3 | *Sphingomonas* strain 2 (1.0×10^4^ cells g^-1^ dry soil) and *Pseudomonas* strain 3 (1.0×10^4^ cells g^-1^ dry soil) |
| *Sph*2*+Pse*4 | *Sphingomonas* strain 2 (1.0×10^4^ cells g^-1^ dry soil) and *Pseudomonas* strain 4 (1.0×10^4^ cells g^-1^ dry soil) |
| *Sph*2*+Pse*5 | *Sphingomonas* strain 2 (1.0×10^4^ cells g^-1^ dry soil) and *Pseudomonas* strain 5 (1.0×10^4^ cells g^-1^ dry soil) |
| *Sph*3*+Pse*1 | *Sphingomonas* strain 3 (1.0×10^4^ cells g^-1^ dry soil) and *Pseudomonas* strain 1 (1.0×10^4^ cells g^-1^ dry soil) |
| *Sph*3*+Pse*2 | *Sphingomonas* strain 3 (1.0×10^4^ cells g^-1^ dry soil) and *Pseudomonas* strain 2 (1.0×10^4^ cells g^-1^ dry soil) |
| *Sph*3*+Pse*3 | *Sphingomonas* strain 3 (1.0×10^4^ cells g^-1^ dry soil) and *Pseudomonas* strain 3 (1.0×10^4^ cells g^-1^ dry soil) |
| *Sph*3*+Pse*4 | *Sphingomonas* strain 3 (1.0×10^4^ cells g^-1^ dry soil) and *Pseudomonas* strain 4 (1.0×10^4^ cells g^-1^ dry soil) |
| *Sph*3*+Pse*5 | *Sphingomonas* strain 3 (1.0×10^4^ cells g^-1^ dry soil) and *Pseudomonas* strain 5 (1.0×10^4^ cells g^-1^ dry soil) |
| *Sph*4*+Pse*1 | *Sphingomonas* strain 4 (1.0×10^4^ cells g^-1^ dry soil) and *Pseudomonas* strain 1 (1.0×10^4^ cells g^-1^ dry soil) |
| *Sph*4*+Pse*2 | *Sphingomonas* strain 4 (1.0×10^4^ cells g^-1^ dry soil) and *Pseudomonas* strain 2 (1.0×10^4^ cells g^-1^ dry soil) |
| *Sph*4*+Pse*3 | *Sphingomonas* strain 4 (1.0×10^4^ cells g^-1^ dry soil) and *Pseudomonas* strain 3 (1.0×10^4^ cells g^-1^ dry soil) |
| *Sph*4*+Pse*4 | *Sphingomonas* strain 4 (1.0×10^4^ cells g^-1^ dry soil) and *Pseudomonas* strain 4 (1.0×10^4^ cells g^-1^ dry soil) |
| *Sph*4*+Pse*5 | *Sphingomonas* strain 4 (1.0×10^4^ cells g^-1^ dry soil) and *Pseudomonas* strain 5 (1.0×10^4^ cells g^-1^ dry soil) |

**Table S4. Detailed inoculation treatments of mesocosm experiment 4.**

| Treatment name | Inoculum |
| --- | --- |
| Ctrl | No microbe |
| *C. lenta* | *Cercomonas lenta* strain ECO-P-01 (1.0×10^2^ cells g-1 dry soil) |
| *Pse*1*+Sph*3+*Chi*1 | *Pseudomonas* strain 1 (1.0×10^4^ cells g^-1^ dry soil), *Sphingomonas* strain 3 (1.0×10^4^ cells g^-1^ dry soil) and *Chitinophaga* strain 1 (1.0×10^4^ cells g^-1^ dry soil) |
| *C. lenta* +*Pse*1*+Sph*3+*Chi*1 | *Pseudomonas* strain 1 (1.0×10^4^ cells g^-1^ dry soil), *Sphingomonas* strain 3 (1.0×10^4^ cells g^-1^ dry soil), *Chitinophaga* strain 1 (1.0×10^4^ cells g^-1^ dry soil) and *Cercomonas lenta* strain ECO-P-01 (1.0×10^2^ cells g-1 dry soil) |

**Table S5. The primers of *gcd and pqq* C genes.**

| Gene_name | Encoded protein | Classification or  Functionality | Forward sequence | Reverse sequence |
| --- | --- | --- | --- | --- |
| gcd | quinoprotein glucose dehydrogenase | Inorganic P solubilization | ATCGCGTTCGGGCCGGACG | ATSAGRTTSAGCTCGTCCCA |
| pqq C | pyrroloquinoline-quinone synthase | Inorganic P solubilization | AACCGCTTCTACTACCAG | GCGAACAGCTCGGTCAG |

**Supplementary materials and methods**

**Isolation and identification of *C. lenta* ECO-P-01**

*C. lenta* ECO-P-01 was isolated from a clay soil in the Netherlands. For this isolation one gram of soil samples was shaken with 20 mL of sterile Page's amoeba saline solution for 30 minutes using a tabletop shaker. Subsequently, one microliter aliquots of the soil suspension were transferred into separate wells of a sterile 96-well plate (Costar, Corning, New York, USA) that contained *Escherichia coli* OP50 as the sole food source. After several days of incubation at 15 °C, each well was screened to select protists under an inverted microscope Nikon Eclipse TS100-F (NIKON, Tokyo, Japan). Wells containing potentially pure protist strains were further diluted several times in order to purify a single protistan strain. The DNA of the purified protistan strain was extracted by the E.Z.N.A Bacterial DNA extraction Kit (Omega, Bio-Tek Inc., Georgia, USA) and the DNeasy Blood & Tissue Kit (QIAGEN N.V., Maryland, USA), following the manufacturer's instructions. To obtain an almost complete 18S rRNA gene sequences of the protistan strain, multiple sets of universal eukaryotic primers were employed (Primer sets: Pre_3ndfor and V4_1rev, 3NDfor and 12Nrev, RibA and RibB, Euk1A_18Sfor and Euk1A_18Srev; for more details about primers sequences see (Gao, 2020), Chapter 3, Supplementary Table 1). The taxonomic classification of the protist strain was determined by conducting a BLASTn search against the NCBI GenBank database.

**Growth condition and preparation of *C. lenta* ECO-P-01**

The pure protistan strain (*C. lenta* ECO-P-01) was maintained in a sterile tube using sterile Page's amoeba saline at 15 °C in our lab (The tube was sealed with Parafilm M tape to prevent pollution by exogenous microorganisms). Inactivated *Escherichia coli* DH5α material was added to the tube every 30 days to maintain protist activity. One microliter of sterile Page's amoeba saline, which contained the pure *C. lenta* ECO-P-01, was pipetted into a sterile polystyrene tissue culture bottle filled with sterile Page's amoeba saline. The bottle contains inactivated *Escherichia coli* DH5α as the sole food source for the growth of *C. lenta* ECO-P-01. *C. lenta* ECO-P-01 was cultivated in a sterile tissue culture bottle in the dark at 15 °C for 5 days. The bottle was sealed with Parafilm M tape to prevent pollution by exogenous microorganisms. To remove remaining inactivated *Escherichia coli* DH5α and obtain axenic protistan cultures, the protistan cultures were centrifuged at 800 g for 5 minutes, and then 75% of the volume was discarded (Gao, 2020). We used an equal volume of sterile Page's amoeba saline to resuspend and wash the protistan cells. This process was repeated five times. After that, we used an inverted microscope Nikon Eclipse Ts2 (objective: Nikon CFI Achromat LWD ADL 40X, Ph1, eyepiece: Nikon TS2-W 10X) (NIKON, Tokyo, Japan) to count the numbers of active *C. lenta* ECO-P-01 individuals for use in the mesocosm experiments.

**Isolation and identification of** **bacterial strains**

The supernatant of homogenized rhizosphere soils was serially diluted using MS buffer solution (10 mM MgSO_4_, 100 mM NaCl, 50 mM Tris-HCl, 0.01% gelatine). Then, 150 μl of the diluted rhizosphere soil suspensions were plated on 1/10 tryptone soy agar (TSA) plates and the TSA plates were placed in an incubator at 30 °C for 48 hours. We randomly selected bacterial isolates and transferred each isolate to a new TSA plate using sterile toothpicks. These TSA plates were placed in an incubator at 30 °C for 2 days. After that, each bacterial isolate was streaked onto a new TSA plate for colony purification. TIANamp Genomic DNA Kits (TIANGEN BIOTECH, China) were used to extract the DNA form the pure bacterial isolates, according to the manufacturer's instructions. The 16S rRNA gene of the bacterial isolates was amplified using the primer set 27F/1492R, and the PCR products were subjected to Sanger sequencing at Tsing Ke Biotech Co., Ltd. (Wuhan, China). To obtain detailed taxonomic information, the full length 16S rRNA gene sequences of the axenic bacterial isolates were subjected to BLAST searches against the NCBI GenBank database.

**Processes of mesocosm experiments**

The inoculum (microbial cells) for each treatment of all mesocosm experiments was suspended in 5 ml of sterilized page's amoeba saline (PAS) (Thomas et al., 2006). This mixed solution was inoculated two days after transplanting cucumber seedlings. After all mixed solutions had been inoculated, sterile distilled H_2_O was added to the soils to approximately 40% soil moisture. Sterile distilled H_2_O was used to water cucumber seedlings every day. Cucumber plants and rhizosphere soil samples of all mesocosm experiments were collected two weeks after inoculation of the solutions. Cucumber plants and rhizosphere soils were collected as described in (Guo et al., 2021, 2022). In brief, cucumber plants were gently pulled out of the soil and shaken vigorously to remove the soil that was not tightly attached to the roots. The shoots were oven-dried at 65 °C for 5 days before the dry biomass was measured. The roots were then placed into a 50 ml centrifuge tube with 40 ml of sterile water and shaken vigorously on a rotary shaker (ZQZY-70B, Zhichu, China) at a speed of 170 r/min for 20 minutes. The roots were removed from the centrifuge tube with sterile tweezers. After that, the centrifuge tube was centrifuged (5810R, Eppendorf, Germany) at a speed of 7168 x g for 15 minutes (4 °C). The resulting soil pellet in the centrifuge tube was defined as the rhizosphere soil. All rhizosphere soil samples were frozen (−80 °C) until further use.

**Plant growth-promoting traits of selected bacterial strains**

The inorganic P solubilization of the selected bacterial strains was quantified in 100 ml of inoculum at 180 rpm and 30 °C for 5 days (rotary shaker: ZQZY-70B, Zhichu, China). The bacterial strains were cultivated in sterile TSB medium to obtain enough cells for subsequent experiments. The bacterial cells were centrifuged (5000 rpm for 10 min at 4 °C) and then resuspended in Pikovskaya (PKO) liquid medium. The suspensions of bacteria were adjusted to a density of 1 ×10^8^ cells/ml by dilution in PKO liquid medium. The inoculum volumes were 2 ml of bacterial suspensions (2 ml of bacterial strain A for monoculture assays, or 2 ml of bacterial strain B for monoculture assays, or 1 ml of bacterial strain A+B ml of bacterial strain 2 for coculture assays) and 98 ml Pikovskaya (PKO) liquid medium. After 5 days, the inoculum was centrifuged at 8000 rpm for 10 min (centrifuge: 5810R, Eppendorf, Germany), and the amount of soluble P in the supernatant was measured by the molybdenum–antimony resistance colorimetric method (Bao, 2000).

The K solubilization of the selected bacterial strains was quantified in 50 ml of inoculum at 150 rpm and 28 °C for 7 days (rotary shaker: ZQZY-70B, Zhichu, China). The bacterial strains were cultivated in sterile TSB medium to obtain enough cells for subsequent experiments. The bacterial cells were centrifuged (5000 rpm for 10 min at 4 °C) and then resuspended in Aleksandrov liquid medium. The suspensions of bacteria were adjusted to a density of 1×10^8^ cells/ml by dilution in Aleksandrov liquid medium. The inoculum volumes were 2 ml of bacterial suspensions (2 ml of bacterial strain A for monoculture assays, 2 ml of bacterial strain B for monoculture assays, or 1 ml of bacterial strain A+1 ml of bacterial strain B for coculture assays) and 49 ml of Aleksandrov liquid medium. The Aleksandrov liquid medium contained K-feldspar powder. After 7 days, the inoculum was centrifuged at 5000 rpm for 10 min (centrifuge: 5810R, Eppendorf, Germany) to measure the K content in the supernatant using flame spectrophotometry (Bao, 2000).

The N_2_ fixation of the selected bacterial strains was quantified in 44 ml of inoculum at 180 rpm and 28 °C for 2 h (in the dark) (rotary shaker: ZQZY-70B, Zhichu, China). The bacterial strains were cultivated in sterile TSB medium to obtain enough cells for subsequent experiments. The bacterial cells were centrifuged (5000 rpm for 10 min at 4 °C) and then resuspended in sterile normal saline. The suspensions of bacteria were adjusted to a density of 1 ×10^7^ cells/ml by dilution in sterile normal saline. The inoculum volumes were 40 ml of bacterial suspensions (40 ml of bacterial strain A for monoculture assays, 40 ml of bacterial strain B for monoculture assays, or 20 ml of bacterial strain A+20 ml of bacterial strain B for coculture assays) and 4 ml of ^15^N_2_ tracer-enriched assay. The ^15^N_2_ tracer-enriched assay medium was prepared according to previously described protocols (Bellenger et al., 2014). Ten percent of the air in the glass bottle (containing the inoculum) was replaced with high-purity ^15^N_2_. After that, the bacterial cells were collected on a Whatman glass microfiber filter (GF/F) (GE Healthcare) and dried. After weighing and grinding, the ^15^N content of the bacterial cells was analyzed by an elemental analyzer-isotope ratio mass spectrometer (EA-IRMS) (Flash 2000-Delta V advantage, Thermo Fisher Scientific).

The ACC deaminase activity of the selected bacterial strains was quantified in 50 ml of inoculum at 180 rpm and 28 °C for 1 day (rotary shaker: ZQZY-70B, Zhichu, China). The bacterial strains were cultivated in sterile TSB medium to obtain enough cells for subsequent experiments. The bacterial cells were centrifuged (5000 rpm for 10 min at 4 °C) and then resuspended in SMA liquid medium. The suspensions of bacteria were adjusted to a density of 1 ×10^8^ cells/ml by dilution in SMA liquid medium. The inoculum volumes were 2 ml of bacterial suspensions (2 ml of bacterial strain A for monoculture assays, 2 ml of bacterial strain B for monoculture assays, or 1 ml of bacterial strain A+1 ml of bacterial strain B for coculture assays) and 48 ml of SMA liquid medium. After 1 day, the inoculum was centrifuged at 8000 rpm and 4 °C for 5 min (centrifuge: 5810R, Eppendorf, Germany). Bacterial cells were washed with Tris-HCL buffer (0.1 mol/l) and resuspended in 600 µl of Tris-HCL buffer (0.1 mol/l). The supernatant was mixed with 30 µl of toluene (Sigma, United States), and the cells were disrupted by vortexing at maximum speed (Genie 2, Scientific Industries, United States) for 1 min. Afterward, 200 µl of the crude enzyme solution was mixed with 20 µl of ACC solution (0.5 mol/l) and placed in water at 30 °C for 15 min. The solution was mixed with 1 ml of HCL (0.56 mol/l) and centrifuged at 10000 rpm for 5 min. Then, 1 ml of the supernatant was mixed with 800 µl of HCl (0.56 mol/l). The solution was incubated at 30 °C for 30 min by adding 300 µl of 2,4-dinitrophenylhydrazine (Sigma, United States). After adding 2 ml of NaOH (2 mol/l), the absorbance of the solution was recorded at 540 nm by spectrophotometry (microplate reader: Synergy H1, BioTek, United States). We used the standard curve of known concentrations of a-butyric acid to estimate the amount of a-butyric acid. One unit enzyme activity (U) was defined as the amount of the substance that catalyzed the ACC to produce a-butyric acid by ACC deaminase per minute. The protein content was measured using the Coomassie Brilliant Blue G-250 method (Bradford, 1976). ACC deaminase activity was defined as the ratio of the unit enzyme activity to total protein content (U/mg).

The IAA production of the selected bacterial strains was quantified in 100 ml of inoculum at 180 rpm and 28 °C for 3 days (rotary shaker: ZQZY-70B, Zhichu, China). The bacterial strains were cultivated in sterile TSB medium to obtain enough cells for subsequent experiments. The bacterial cells were centrifuged (5000 rpm for 10 min at 4 °C) and then resuspended in yeast malt dextrose broth liquid medium. The suspensions of bacteria were adjusted to a density of 1×10^8^ cells/ml by dilution in yeast malt dextrose broth liquid medium. The inoculum volumes were 2 ml of bacterial suspensions (2 ml of bacterial strain A for monoculture assays, 2 ml of bacterial strain B for monoculture assays, or 1 ml of bacterial strain A+1 ml of bacterial strain B for coculture assays) and 98 ml of yeast malt dextrose broth liquid medium. After 3 days, the inoculum was centrifuged at 5000 rpm for 10 min (centrifuge: 5810R, Eppendorf, Germany). The supernatant was stored, and 1 ml of the supernatant was mixed with 2ml of Salkowski's reagent and kept it in the dark. After 120 min, the absorbance of the solution was recorded at 530 nm by spectrophotometry (microplate reader: Synergy H1, BioTek, United States) and we used the standard curve of known concentrations of IAA to estimate the amount of IAA in the solution.

The siderophore production of the selected bacterial strains was quantified in 100 ml of inoculum shaken at 180 rpm and 30 °C for 72 h (rotary shaker: ZQZY-70B, Zhichu, China). The bacterial strains were cultivated in sterile TSB medium to obtain enough cells for subsequent experiments. The bacterial cells were centrifuged (5000 rpm for 10 min at 4 °C) and then resuspended in LB liquid medium. The suspensions of bacteria were adjusted to a density of 1 ×10^8^ cells/ml by dilution in LB liquid medium. The inoculum volumes were 2 ml of bacterial suspensions (2 ml of bacterial strain A for monoculture assays, 2 ml of bacterial strain B for monoculture assays, or 1 ml of bacterial strain A+1 ml of bacterial strain B for coculture assays) and 98 ml of LB liquid medium. After 72 h, the inoculum was centrifuged at 6000 rpm for 10 min (centrifuge: 5810R, Eppendorf, Germany). The supernatant was mixed with Chrome Azurol Sulphonate (CAS) liquid medium at a ratio of 1:1. The siderophore content was calculated using the following formula: SU=(Ar-As)/Ar, in which Ar is the absorbance of the non-bacterial group as a reference and As is the absorbance of the sample at 630 nm (microplate reader: Synergy H1, BioTek, United States).

The ammonia production of the selected bacterial strains was quantified in 10 ml of inoculum at 180 rpm and 30 °C for 2 days (rotary shaker: ZQZY-70B, Zhichu, China). The bacterial strains were cultivated in sterile TSB medium to obtain enough cells for subsequent experiments. The cells of bacteria were centrifuged (5000 rpm for 10 min at 4 °C) and then resuspended in 1% peptone water. The bacterial suspensions were adjusted to a density of 1×10^8^ cells/ml by dilution in 1% peptone water. The inoculum volumes were 2 ml of bacterial suspensions (2 ml of bacterial strain A for monoculture assays, 2 ml of bacterial strain B for monoculture assays, or 1 ml of bacterial strain A+1 ml of bacterial strain B for coculture assays; concentration:1 ×10^8^ cells/ml) and 8 ml of 1% peptone water. After 2 days, the inoculum was centrifuged at 6000 rpm for 10 min (centrifuge: 5810R, Eppendorf, Germany) and 2 ml of the supernatant was mixed with 0.5 ml of Nessler's reagent. The absorbance of the solution was recorded at 450 nm by spectrophotometry (microplate reader: Synergy H1, BioTek, United States). We used the standard curve of known concentrations of ammonium sulfate to estimate the amount of ammonia in the solution.

**Biofilm formation of selected bacterial strains and the predatory protist *C. lenta* in monoculture and coculture systems**

Bacterial strains and the predatory protist *C. lenta* were cultivated in sterile tryptic soy broth (TSB) medium and PAS to obtain enough cells for subsequent experiments, respectively. The cells of bacteria and protist were centrifuged (5000 rpm for 10 minutes at 4 °C) and then resuspended in TSB medium. The suspensions of bacteria and protists were adjusted to a density of 1×10^6^ cells ml^-1^ and a density of 1 ×10^4^ cells ml^-1^, respectively, by dilution in TSB medium. The biofilms of the monoculture and coculture systems were cultivated in 200 µl inoculum at 30 °C for 3 days on Nunc-TSP plates. The inoculum volumes were as follows: (1) 40 µl of bacterial strain A+160 µl of TSB medium for monoculture assays, (2) 40 µl of bacterial strain B+160 µl of TSB medium for monoculture assays, (3) 20 µl of bacterial strain A+20 µl of bacterial strain B+160 µl of TSB medium for coculture assays, (4) 40 µl of *C. lenta*+160 µl of TSB medium for monoculture assays, (5) 40 µl of bacterial strain A+40 µl of *C. lenta*+120 µl of TSB medium for coculture assays, (6) 40 µl of bacterial strain B+40 µl of *C. lenta*+120 ul of TSB medium for coculture assays, (7) 20 µl of bacterial strain A+20 µl of bacterial strain B+40 µl of *C. lenta*+120 µl of TSB medium for coculture assays. After 3 days of incubation at 30 °C, we quantified biofilm formation in the monoculture and coculture systems using a modified crystal violet (CV) assay.

References:

1. Gao Z. Soil protists: from traits to ecological functions. 2020. Utrecht University.

2. Thomas V, Herrera-Rimann K, Blanc DS, Greub G. Biodiversity of amoebae and amoeba-resisting bacteria in a hospital water network. Appl Environ Microbiol 2006; 72: 2428–2438.

3. Guo S, Tao C, Jousset A, Xiong W, Wang Z, Shen Z, et al. Trophic interactions between predatory protists and pathogen-suppressive bacteria impact plant health. ISME J 2022; 16: 1932–1943.

4. Guo S, Xiong W, Hang X, Gao Z, Jiao Z, Liu H, et al. Protists as main indicators and determinants of plant performance. Microbiome 2021; 9: 64.

5. Bao SD. Soil agriculture and chemical analysis. 2000. China Agriculture Press, Beijing, China.

6. Bellenger JP, Xu Y, Zhang X, Morel FM, Kraepiel AML. Possible contribution of alternative nitrogenases to nitrogen fixation by asymbiotic N_2_-fixing bacteria in soils. Soil Biol Biochem 2014; 69: 413–420.

7. Bradford MM. A rapid and sensitive method for the quantitation of microgram quantities of protein utilizing the principle of protein-dye binding. Anal Biochem 1976; 72: 248–254.
